# Rice protein phosphatase 1 regulatory subunits OsINH2 and OsINH3 participate actively in growth and adaptive responses under abscisic acid

**DOI:** 10.3389/fpls.2022.990575

**Published:** 2022-09-07

**Authors:** Sawaira Jadoon, Qianqian Qin, Weiqiang Shi, Yan Longfeng, Suiwen Hou

**Affiliations:** Ministry of Education Key Laboratory of Cell Activities and Stress Adaptations, School of Life Sciences, Lanzhou University, Lanzhou, China

**Keywords:** PP1, OsINH2, OsINH3, rice growth, ABA, ROS

## Abstract

Rice (*Oryza sativa* L.), a worldwide staple food crop, is affected by various environmental stressors that ultimately reduce yield. However, diversified physiological and molecular responses enable it to cope with adverse factors. It includes the integration of numerous signaling in which protein phosphatase 1 (PP1) plays a pivotal role. Research on PP1 has been mostly limited to the PP1 catalytic subunit in numerous cellular progressions. Therefore, we focused on the role of PP1 regulatory subunits (PP1r), *OsINH2* and *OsINH3*, homologs of *AtINH2* and *AtINH3* in Arabidopsis, in rice growth and stress adaptations. Our observations revealed that these are ubiquitously expressed regulatory subunits that interacted and colocalized with their counter partners, type 1 protein phosphatase (OsTOPPs) but could not change their subcellular localization. The mutation in *OsINH2* and *OsINH3* reduced pollen viability, thereby affected rice fertility. They were involved in abscisic acid (ABA)-mediated inhibition of seed germination, perhaps by interacting with osmotic stress/ABA-activated protein kinases (OsSAPKs). Meanwhile, they positively participated in osmotic adjustment by proline biosynthesis, detoxifying reactive oxygen species (ROS) through peroxidases (POD), reducing malondialdehyde formation (MDA), and regulating stress-responsive genes. Moreover, their co-interaction proposed they might mediate cellular processes together or by co-regulation; however, the special behavior of two different PP1r is needed to explore. In a nutshell, this research enlightened the involvement of *OsINH2* and *OsINH3* in the reproductive growth of rice and adaptive strategies under stress. Hence, their genetic interaction with ABA components and deep mechanisms underlying osmotic regulation and ROS adjustment would explain their role in complex signaling. This research offers the basis for introducing stress-resistant crops.

## Introduction

Rice (*Oryza sativa* L.) is a globally important cash crop, feeding half of the world’s population and serving 80% sources of calories (Agarwal et al., 2016). However, multiple detrimental factors influence rice crops together and lead to remarkable production loss (Dolferus, 2014; Reddy et al., 2017). Up to 70% yield loss occurs due to various stressors which affect rice growth, seed development, and potential survival (Akram et al., 2019). Despite the deleterious effect of environmental factors, plants can survive by evolving numerous cellular and molecular mechanisms.

Complex pathways are involved in the perception and transduction of stress signals, among which key events are phosphorylation and dephosphorylation. In protein phosphorylation, kinase proteins play an active role, while the reverse reaction is catalyzed by protein phosphatases (You et al., 2014). Reversible protein phosphorylation is pivotal for the posttranslational regulation of cellular and developmental processes (Luan, 2003). In dephosphorylation, one potential player is protein phosphatase 1 (PP1). It belongs to the Ser/Thr phosphoprotein phosphatase family (Shi, 2009) and is strongly conserved among eukaryotes (Cohen, 2002). PP1 comprises a catalytic subunit (PP1c) and varied regulatory subunits (PP1r) that bind to specific targets, recruit PP1c, and define its catalytic activity and location in the cell (Bollen, 2001). The PP1r possesses an active site, consisting of a hydrophobic sequence (RVXF motif) for substrate recruitment and cellular targeting (Verbinnen et al., 2017). The PP1c is an approximately 37-kD protein (Lee et al., 1999) that has been studied in plants extensively, whereas, the identification and functioning of PP1r in plants, especially in crops, are neglected.

In mammals, about 140 PP1 regulatory subunits have been found with diverse functions (Hendrickx et al., 2009). These proteins exist as inhibitor proteins and were originally identified as heat-stable proteins (Brandt et al., 1975). The earliest discovered inhibitor proteins are Inhibitor-1(I-1, also named INH1; Connor et al., 1998), DARPP-32 (Svenningsson et al., 2004), Inhibitor-2 (INH2; Bollen and Stalmans, 1992), and Inhibitor-3 (INH3; Zhang et al., 1998). The INH2, identified from rabbit skeletal muscle, is a highly conserved protein that can inhibit phosphatase activity (Leach et al., 2003). The biochemical and sequence analysis reveals it contains four potential fragments including PXTP, [SG] ILK, RVXF, and αhelical (Hurley et al., 2007). Although it was identified 30 years ago, to date its role in plants is poorly understood. It has been reported in Arabidopsis as an inhibitor of Type One Protein Phosphatase’s (TOPP) catalytic activity (Templeton et al., 2011) and also as a negative regulator of abscisic acid (ABA) signaling by interacting with TOPPs and sucrose non-fermenting 1-related protein kinases 2 (SnRK2.6; Hou et al., 2016). Likewise, the contribution of INH3, a small nuclear protein of 126 amino acids, was just reported in embryo development and inhibition of PP1c catalytic activity (Takemiya et al., 2009). Recently, our lab identified another PP1 regulatory subunit, Protein Phosphatase 1 Regulatory Subunit 3 (PP1R3) in Arabidopsis, which regulates ABA signaling as a holoenzyme with TOPPs (Zhang et al., 2020). Besides, another putative regulatory subunit of PP1, PP1 Regulatory Subunit 2-like Protein 1 (PRSL1) has also been studied as a positive modulator of stomatal opening and blue light signaling in *Vicia faba* (Takemiya et al., 2006; Shimazaki et al., 2007). However, the role of PP1r in crops, particularly in reproductive growth, osmotic adjustment and homeostasis of reactive oxygen species (ROS) are still unknown. Therefore, we decided to explore the roles of PP1r, OsINH2, and OsINH3 in rice growth and responses to ABA.

Plants have evolved multiple mechanisms and signaling routes for their better survival. The participation of PP1c has been explored in various signaling, such as TOPP4 regulating DELLA-mediated gibberellic acid (GA) signaling, pavement cell morphogenesis by Pin-formed 1 (PIN1), and light morphogenesis by modulating Phytochrome Interacting Factor5 (PIF5; Qin et al., 2014; Guo et al., 2015; Yue et al., 2015). Moreover, TOPPs were studied as holoenzymes with Pi0431 in the promotion of late blight disease (Boevink et al., 2016). TOPPs also contribute to plant immunity by interacting with suppressors of topp4-1 (SUT1; Yan et al., 2019; Liu et al., 2020) and reduce water soaking and disease susceptibility *via* ABA (Hu et al., 2022). By contrast, the study on plant’s PP1r in signaling pathways is limited, thus we paid attention to the activity of OsINH2 and OsINH3 in the ABA signaling cascade.

The ABA, a foremost important stress hormone, regulates various aspects of plant growth under unfavorable conditions and enables it to survive potentially. It activates one of the signaling components, SnRK2s, which further phosphorylate downstream substrates, induce expression of ABA-responsive genes and stimulate other responses (Fujii and Zhu, 2009). In rice, 10 members of the SnRK2s have been identified termed as osmotic stress/ABA-activated protein kinases (SAPK1-10), which are involved in numerous growth responses (Umezawa et al., 2013). For instance, SAPK6 shows ABA insensitivity in tobacco during seed germination and root elongation (Chae et al., 2007) while SAPK2 is resistant to ABA in rice (Lou et al., 2017). The SAPK1 and SAPK2 participate in seed germination and seedling growth under NaCl (Lou et al., 2018); similarly, SAPK4 confers salt resistance in rice (Diédhiou et al., 2008). While the SAPK9 positively regulates the ABA-mediated signaling pathway in rice under drought (Dey et al., 2016). Whereas, SAPK10 stimulates jasmonic acid (JA) biosynthesis in the presence of ABA and inhibits seed germination (Wang et al., 2020).

Besides growth, ABA also regulates ROS, which act downstream of the ABA signaling pathway (Postiglione and Muday, 2020). The ROS generation is definite in higher plants during normal metabolism. However, ROS imbalance by various stressors can disrupt the normal functioning of a cell by damaging proteins, nucleic acid, and lipids (Mittler, 2006). Therefore, plants adopt integrated strategies including physiological changes, osmotic adjustment, ionic homeostasis, solute production, and activation of an antioxidant defense system, in which ABA plays a key role (Wei et al., 2015; Guajardo et al., 2016) For instance, upon ROS accumulation, plants stimulate enzymatic protection strategies including APX ascorbate peroxidase (APX), catalase (CAT), glutathione reductase (GR), super oxidase dismutase (SOD), and peroxidase dismutase (POD) production (Lin and Pu, 2010; Luo et al., 2010; Zhang et al., 2013). As well as, non-enzymatic antioxidants, such as ascorbate (AsA) and glutathione (GSH), also adjust ROS imbalance (Noctor and Foyer, 1998). Previous studies reported that ABA application induces enzymatic and non-enzymatic antioxidants in maize (Jiang and Zhang, 2001). Besides, activation of stress-responsive genes and proline biosynthesis are also adaptive strategies of the plant under unfavorable conditions. The stress-responsive genes regulate the expression of other genes that encode important enzymes and metabolic proteins, while proline participates in osmotic adjustment, and protects proteins, enzymes, and membranes (Anwar Hossain et al., 2014). However, to date, the involvement of PP1r in above-mentioned responses has not been focused.

In summary, this article has described the contribution of OsINH2 and OsINH3, the homolog of Arabidopsis INH2 and INH3, in rice growth and adaptive responses under ABA. As regulatory subunits, they can interact with OsTOPPs and colocalize with them, but could not change their subcellular localization. Our findings revealed that *OsINH2* and *OsINH3* participate in the reproductive growth of rice, regulate ABA-mediated inhibition of seed germination, and contribute to ROS homeostasis. Our study would offer significant grounds for introducing stress-resistant crops.

## Materials and methods

### Plant materials and growth conditions

In this study, we used CRISPR/Cas9 generated knockout lines of *OsINH2* and *OsINH3* in wild type, Zhonghua-11 (ZH11) background. Homozygous mutant lines were screened by PCR-sequencing analysis and HYG-resistance by using genomic primers (Supplementary Table 1). While for *OsINH2* and *OsINH3* overexpression lines, their CDS was cloned into pOx vector driven by maize ubiquitin gene promoter and then transferred into ZH11. Plants were grown in a greenhouse with a 12 h light (28–30°C)/12 h dark (20–22°C) cycle with 60% humidity.

### Growth observation and pollen morphology

All genotypes were keenly observed from seed germination up to harvesting. Reproductive traits such as pollen morphology and fertility were noticed after the heading stage. The pollen grains were observed microscopically after staining with Potassium Iodide (KI). Usually, after KI staining, the normal (viable) round pollen of rice turns dark blue, while non-viable or poorly developed pollen is often deformed, and becomes brown. We prepared KI solution by dissolving 2 g potassium in 5–10 ml ddH_2_O, and then added 1 g Iodine and made the final volume up to 300 ml. To stain pollen grains, we selected fully developed flowers whose anthers were enclosed in florets and burst an anther on a slide in a drop of ddH_2_O, then added 1–2 drops of KI solution and observed under a light microscope. The active and inactive pollen were counted per 100 pollen grains. The seeds were counted per tiller to analyze the fertility of all genotypes.

### Seed germination assay

Seed germination assay was performed as previously reported (Lin et al., 2015) with a few modifications. The seeds of ZH11, *OsINH2,* and *OsINH3* lines were surface sterilized with 70% ethanol for 1 min and immersed in 50% NaOCl for 10 min, and then washed with sterilized water at least five times. Finally rinsed seeds were planted on half MS media without ABA (as mock) and MS supplemented with 5 μm ABA (as treatment). Then plates were placed in a growth chamber having conditions according to Liao et al. (2016). The germination rate was recorded every day for up to 7 days (until ZH11 seeds reached to maximum germination rate), and the relative germination was recorded at 4 days after germination (DAG). The ABA sensitivity of seeds was analyzed by one-way ANOVA.

### Post germination growth assay and stress treatment

For post-germination, seeds germinated on half MS and 1 week old seedlings were transferred into nutrient solution as mentioned previously (Wang et al., 2014a). The transcript level of various genes including *OsINH2*, *OsINH3*, *OsNAC1*, *OsLEA3*, *OsLIP19*, *and OsP5CS1*, was measured at the three-leaf stage. Moreover, to check ROS and antioxidant enzymes, we followed the method described by You et al. (2014). Three leaf staged seedlings were sprayed with 100 μM ABA. After completion of the experiment duration, leaves were sampled, immediately frozen in liquid nitrogen, and stored at −80°C for further experiments.

### Histochemical detection of GUS activity and subcellular localization

β-Glucuronidase (GUS) staining was described previously (Qin et al., 2014). The promoter sequences of *OsINH2* and *OsINH3* (3.3 and 3 kb), respectively, were inserted into the pCAMBIA1301 GUS plasmid. After transformation to ZH11, different tissues of GUS transgenic plants (*ProOsINH2: GUS/* ZH11 *and ProOsINH2: GUS/*ZH11) were stained by using GUS working solution for one night at 37°C. Then decolorize with 70% ethanol and imaged using a LEICA (M205A) stereomicroscope. In addition, for cellular localization, YFP-OsINH2 and YFP-OsINH3 were applied to tobacco leaves and fluorescence signals were observed by using a laser microscope (Zeiss).

### Quantitative RT-PCR analysis

To detect the expression profile of required genes in respective plant materials, we extracted total mRNA and converted it into cDNA according to Wang et al. (2014b). The cDNA was amplified by using specific qRT-PCR primers (Supplementary Table 2). For detection of transcriptional changes under NaCl and ABA stress, three leaf stage rice seedlings were used after treatment for a specific duration. The enzyme, SYBRPremix Ex Taq (Takara Bio) was used for amplification in ABI StepOnePlus™ Real-Time PCR Systems (Thermo Fisher Scientific). The *Ubiquitin 10* (*UBQ10*) was used as a reference gene.

### Plasmids construction and plant transformation

To construct overexpression lines, the full-length CDS sequence of OsINH2 and OsINH3 was amplified by using primers having a specific enzyme sequence (Kpn1 linked to forward primers and BamH1 with reverse primers). Amplified fragments were inserted into the pOx plasmid by the enzyme digestion method, and the final plasmids were transferred into ZH11. Besides the overexpression vector, we constructed *ProOsINH2: GUS* and *ProINH3: GUS* plasmids by fusing (3.3 and 3 kb) promoter regions of *OsINH2* and *OsINH3,* respectively, with pCAMBIA1301 plasmid. Moreover, we generated plasmids for the Yeast-two-hybridization (Y2H) system. The CDS of OsINH2 and OsINH3 was linked to the Gal4-activation domain (prey protein, pGADT7), while OsTOPPs and OsSAPKs full-length coding sequences were fused to the Gal4 DNA binding domain (bait protein, pGBKT7) by the infusion method. Furthermore, plasmids for biomolecular fluorescence complementation (BiFC) assay were constructed by the GATEWAY system. The CDS of OsINH2 and OsINH3 was linked to YFP (to check cellular localization), while OsTOPPs were fused with RFP (to observe colocalization with regulatory proteins). Additionally, OsINH2 and OsINH3 were fused with the C-terminal half of yellow fluorescent protein (YFP^c^), and OsTOPPs and OsSAPKs joined with the N-terminal of YFP (YFP^n^). In the end, all plasmids were injected *Nicotiana benthamiana* through *agrobacterium* and fluorescence signals were observed through the confocal microscope.

### Yeast-two-hybridization assay

The Y2H analysis was carried out according to Zhang et al. (2020). The yeast containing OsINH2-AD, OsINH3-AD, OsTOPPs-BD, and OsSAPK4-BD plasmids was spread onto DDO (SD/−Leu/−Trp) media plates and incubated at 28°C for 3–4 days. After completion of the incubation period, a single clone for every sample was inoculated on QDO (SD/−Leu/−Trp/-His/−Ade/+ X-a-Gal agar) plates and incubated for 3–7 days. Next, colony morphology was observed.

### Bimolecular fluorescence complementation assay

For the biomolecular fluorescence complementation (BiFC) assay, we followed methods described by Hu et al. (2018). The OsINH2-YFP^c^ and OsINH3-YFPc were co-infiltrated with TOPPs-YFP^n^ and SAPKs-YFP^n^. Moreover, for confirmation of OsINH2 interaction with OsINH3, the OsINH3-YFP^c^ and OsINH3-YFP^n^ were co-expressed in *Nicotiana benthamiana via Agrobacterium*. The fluorescence signals were observed by confocal laser microscope after 3 days of sample injection into leaves.

### Measurement of stress-associated indicators

To determine ROS in all genotypes under normal conditions and after ABA treatment, leaves were stained with nitroblue tetrazolium (NBT) by using the reported method (He et al., 2012). Furthermore, oxidative damage was assessed by quantifying MDA content according to the instruction of the commercial kit (Solarbio, Cat No: BC0020). The free proline content was estimated according to Lou et al. (2017). L-Proline was used to calculate the standard concentration of proline and absorbance was measured at a wavelength of 530 nm by using a spectrophotometer. Besides, the enzymatic activities including POD, SOD, and CAT were estimated by using commercial kits (Solarbio Cat No: BC0090, BC0170, and BC0200), respectively, and followed the instructions of the above-mentioned kits.

### Accession numbers

The accession numbers for *OsINH2*, *OsINH3*, *OsTOPPs,* and *OsSAPKs* are enlisted (Supplementary Table 3).

## Results

### Identification of *OsINH2* and *OsINH3* in rice

The conservative nature of PP1 among eukaryotes is well known. The inhibitor proteins, INH2 and INH3, have already been reported in mammals, yeast, and Arabidopsis. However, in rice (*Oryza sativa*), the activity of these proteins is still elusive. By keeping in view, the conservation of *INH2* and *INH3* in Plantae, we searched their homologs in rice. By blasting the protein sequence of Arabidopsis INH2 and INH3 in the database, National Center for Biotechnology Information (NCBI), we found one OsINH2 protein comprised of 165 amino acids while OsINH3 has two homologs of 13 kDa. The conservation pattern of these inhibitor proteins is alike across Plantae as they also possess RVXF motif like inhibitor proteins in Arabidopsis (Takemiya et al., 2009; Templeton et al., 2011). So, we compared an amino acid sequence of INH2 and INH3 in Arabidopsis and rice (Supplementary Figure 1) and predicted they are likely to have evolutionarily conserved functions in these two species. Therefore, we investigated their roles in rice growth and response to ABA.

### Expression characterization of *OsINH2* and *OsINH3* and their protein subcellular localization

**Figure 1:**
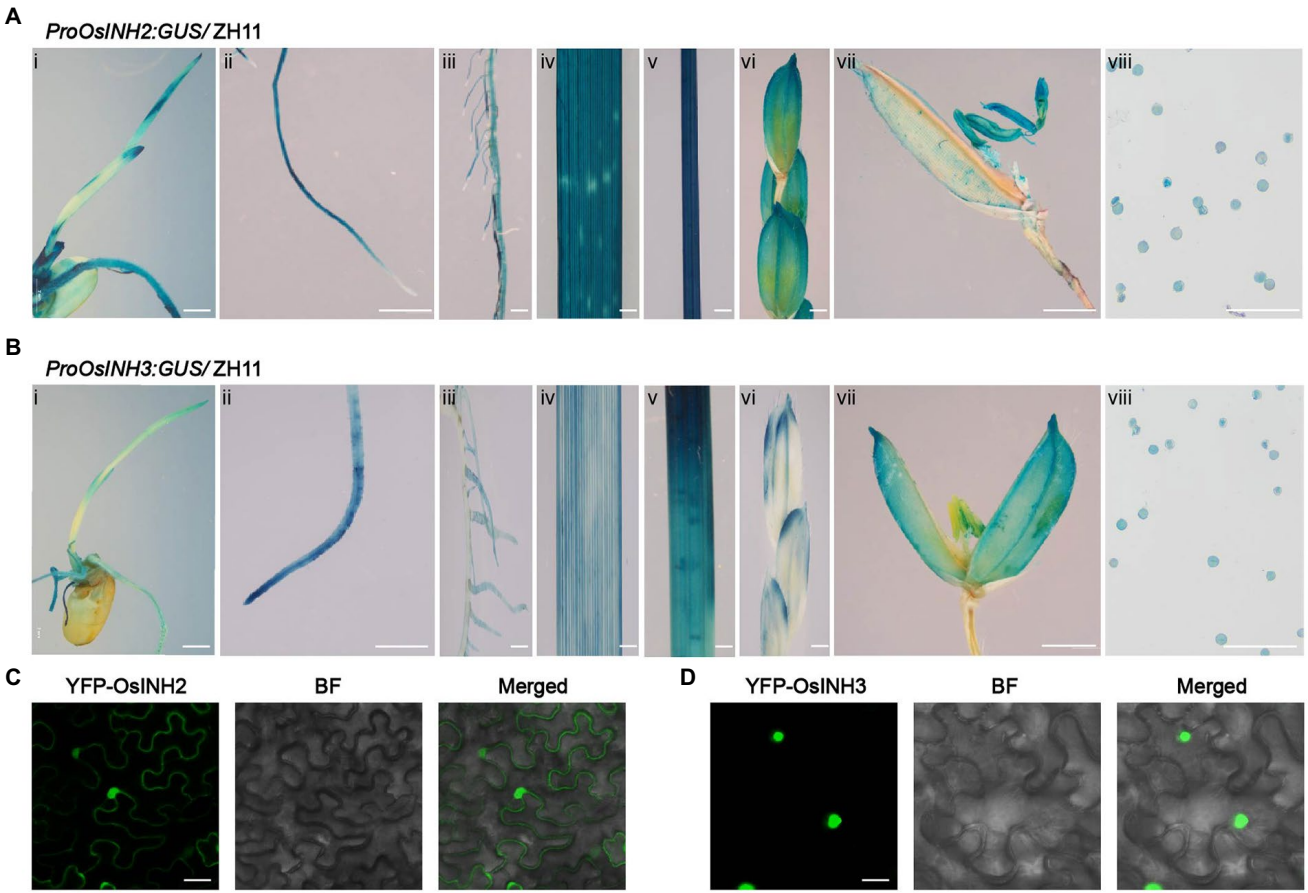
Tissue-specific expression of *OsINH2* and *OsINH3* and their protein subcellular localization. **(A)** Expression patterns of *OsINH2*. **(i)** A 4-day-old seedling, **(ii)** Primary root, **(iii)** Crown roots, **(iv)** Leaf, **(v)** Stem, **(vi)** Florets, **(vii)** Flower, and **(viii)** Pollen grains. **(B)** Expression patterns of *OsINH3*. **(i)** A 4-day-old seedling, **(ii)** Primary root, **(iii)** Crown roots, **(iv)** Leaf, **(v)** Stem, **(vi)** Florets, **(vii)** Flower, and **(viii)** Pollen grains. Scale bars, **(Ai)** and **(Bi)** = 2 mm, **(Aii–vii)** and **(Bii–vii)** = 20 mm, **(Aviii)** and **(Bviii)** = 30 μm. **(C,D)** Subcellular localization of OsINH2 and OsINH3. YFP-OsINH2 and YFP-OsINH3 were injected in *N.benthamiana* leaves. BF, bright field. Scale bars, 25 μm.

To study expression features, the *proOsINH2: GUS/* ZH11 *and proOsINH3: GUS/*ZH11 transgenic plants were used. Histochemical analysis disclosed that both *OsINH2* and *OsINH3* showed ubiquitous expression in rice. The promoter of *OsINH2* and *OsINH3* was highly activated at the germination and post-germination stages as high *GUS* activity was noticed in young seedlings, primary root, crown roots, leaf, and shoot (Figures 1A,B). While, at the reproductive stage, their expression was detected in panicle, anthers, and pollen grains (Figures 1A,B). In addition, consistent with *GUS* activity, the qRT-PCR analysis also showed a noticeable expression of *OsINH2* and *OsINH3* in various tissues (Supplementary Figure 2). Moreover, by focusing on that protein’s functions depend on their localization inside the cell, we studied their subcellular localization. *Agrobacterium* carrying YFP-OsINH2 and YFP-OsINH3 was infiltrated into *Nicotiana benthamiana*. The transient expression analysis declared that the YFP-OsINH2 was localized both in the nucleus and cytoplasm while YFP-OsINH3 fluorescence signals were observed mainly in the nucleus (Figures 1C,D). These results suggest that inhibitor proteins may play redundant functions in rice.

### OsINH2 and OsINH3 interacted and colocalized with OsTOPPs

**Figure 2:**
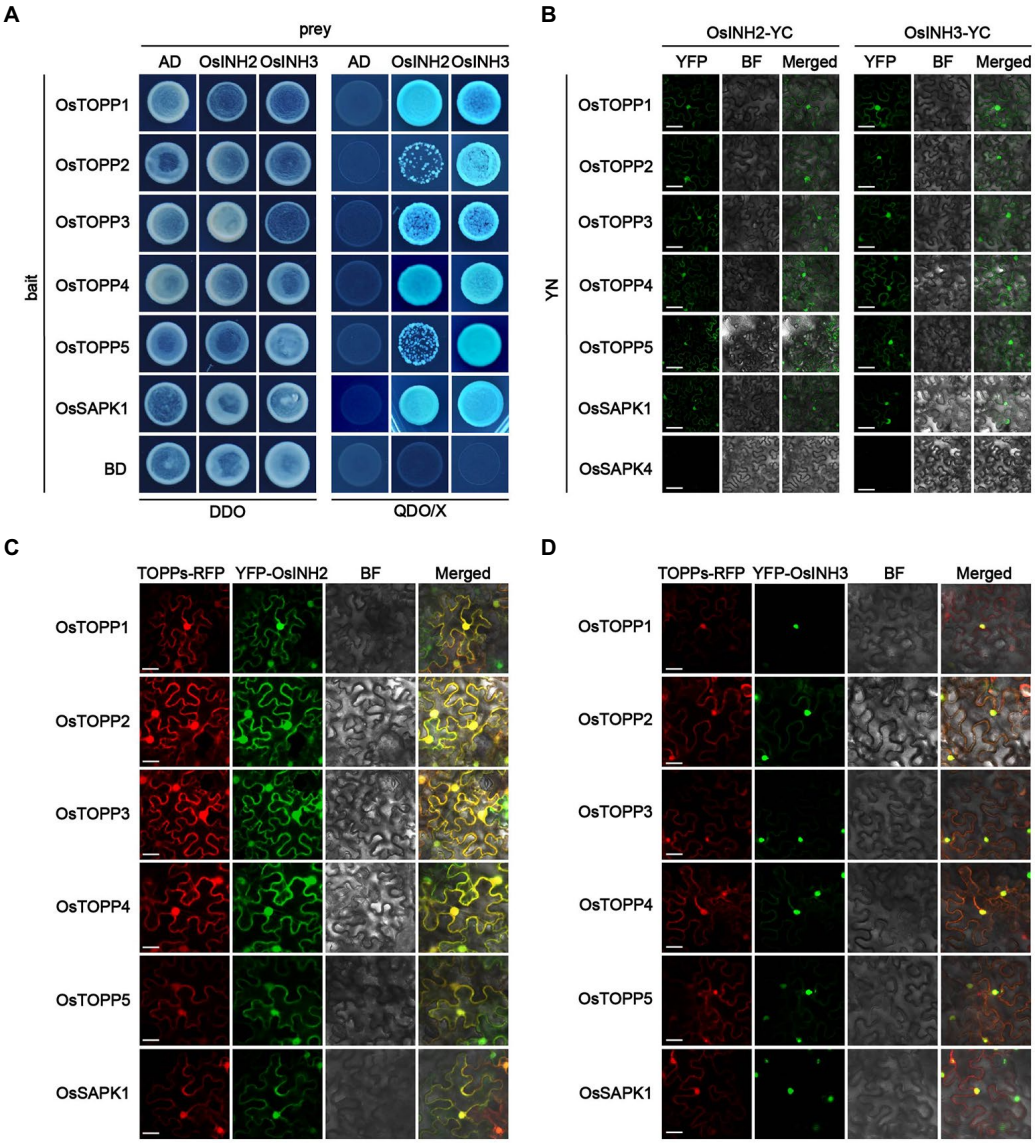
OsINH2 and OsINH3 interacted and colocalized with OsTOPPs. **(A)** Showing the interaction of OsINH2 and OsINH3 with OsTOPPs in the Yeast-two-hybridization (Y2H) system. OsINH2-AD and OsSAPK1-BD or OsINH3-AD and OsSAPK1-BD were used as a positive control. OsINH2 and OsINH3 fused with empty AD and OsTOPPs fused with empty BD were used as negative control. DDO, SD/−Leu/−Trp; QDO/X, SD/−Leu/−Trp/-His/−Ade supplemented with X-α-Gal. **(B)** Biomolecular fluorescence complementation (BiFC) assay representing the interaction of OsINH2 and OsINH3 with OsTOPPs. OsINH2-YC and OsTOPPs-YN or OsINH3-YC and OsTOPPs-YN were co-expressed in *Nicotiana benthamiana* leaves. OsINH2-YC and OsSAPK1-YN or OsINH3-YC and OsSAPK1-YN were used as positive control. OsINH2-YC and OsSAPK4-YN or OsINH3-YC and OsSAPK4-YN were used as negative control. Scale bars, 30 μm. **(C,D)** Transient expression analysis illustrated OsINH2 and OsINH3 colocalized with OsTOPPs *in vivo*. OsTOPPs-RFP and YFP-OsINH2 or OsTOPPs-RFP and YFP-OsINH3 were co-infiltrated in *N. benthamiana* leaves. OsSAPK1-RFP and YFP-OsINH2 or OsSAPK1-RFP and YFP-OsINH3 were used as control. BF, bright field. Scale bars, 25 μm.

As the PP1r could bind to the PP1c and regulates numerous physiological and cellular processes, we confirmed the binding ability of OsINH2 and OsINH3 with OsTOPPs by the Y2H system. As expected, OsINH2 and OsINH3, fused with the Gal4 activation domain (AD) interacted with OsTOPPs linked to the Gal4 DNA binding domain (BD; Figure 2A). To further validate PP1r-PP1c interaction in planta, we performed a BiFC assay in *N.benthamiana* leaves. Co-expression of OsINH2-YFP^c^ and OsINH3-YFP^c^ with OsTOPPs-YFP^n^ showed fluorescence signals in the nucleus and cytoplasm (Figure 2B). By knowing that majority of binding proteins interact with PP1 through the RVXF motif (Moorhead et al., 2007), we constructed OsINH2^V6A/W8A^-AD and OsINH3^V50A/W52A^-AD and checked their interaction with OsTOPPs. The Y2H and BiFC results demonstrated that mutant OsINH2 and OsINH3 also interacted with OsTOPPs (Supplementary Figure 3).

Moreover, from the interaction of PP1r-PP1c, we predicted their colocalization *in vivo*. Co-expression of YFP-OsINH2 or YFP-OsINH3 with OsTOPPs-RFP in *N.benthamiana* leaves indicated OsINH2 colocalized with OsTOPPs in the entire cell (Figure 2C), while OsINH3 colocalization with OsTOPPs was observed mainly in the nucleus (Figure 2D). However, both regulatory proteins have not changed OsTOPP’s localization. Overall, these results suggest that OsINH2 may bind with OsTOPPs in the nucleus and cytoplasm, but OsINH3 bind with OsTOPPs only in the nucleus to play function. Therefore, we predicted that OsINH2 and OsINH3 could play role in growth and stress responses by interacting with OsTOPPs.

### OsINH2 and OsINH3 were involved in the reproductive growth of rice

**Figure 3:**
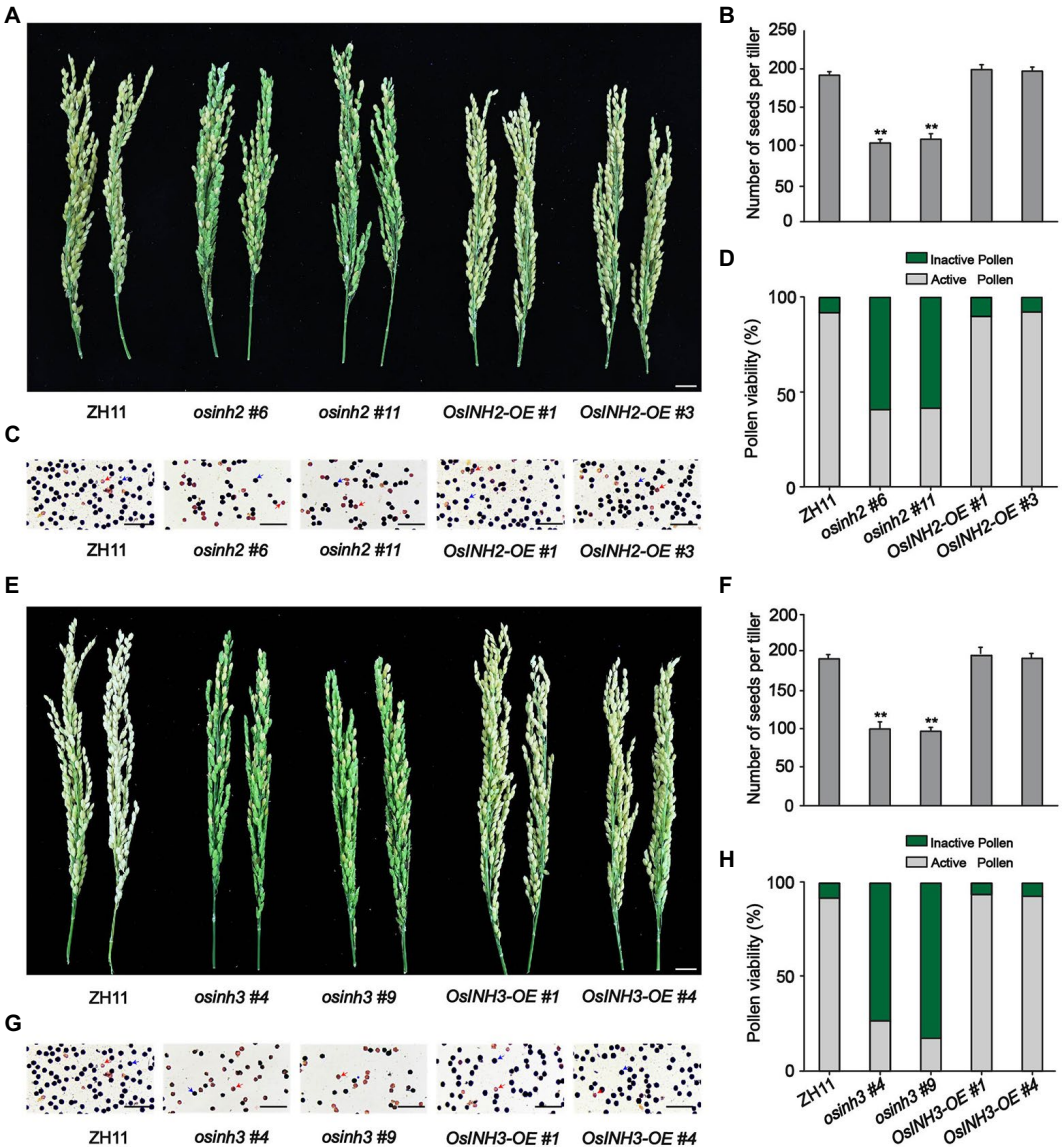
Performance of *OsINH2* and *OsINH3* in rice growth. **(A)** Phenotypes of the spikelet fertility in ZH11 and *OsINH2* genotypes. Scale bar, 2 cm. **(B)** Illustrating the number of seeds in ZH11 and *OsINH2* lines. **(C)** Morphological observation of the pollen grains in ZH11 and *OsINH2* lines. Scale bars, 30 μm. **(D)** Indicating percentage of active and inactive pollen in ZH11 and *OsINH2* genotypes. **(E)** Phenotypes of the spikelet fertility in ZH11 and *OsINH3* genotypes. Scale bar, 2 cm. **(F)** Showing the number of seeds in ZH11 and *OsINH3* genotypes. **(G)** Morphological observation of the pollen grains in ZH11 and *OsINH3* lines. Scale bars, 30 μm. **(H)** Representing the percentage of active and inactive pollen in ZH1 and *OsINH3* genotypes. After KI staining, the normal (viable) round pollen of rice turned dark blue, while non-viable or poorly developed pollen became brown. In **(C,G)**, blue arrows represent active pollen grains (dark blue) and red arrows represent inactive pollen grains (brown). In **(B,F)**, graph bars represent mean data. Error bars indicate ±SE with biological triplicates (*n* = 25), asterisks specify the significant difference between the ZH11 and transgenic lines as determined by Student’s *t*-test analysis, *p < 0.05; ^**^p < 0.01. In **(D,H)** for pollen activity, *n* = 6.

To study the involvement of *OsINH2 and OsINH3* in rice growth, we observed the growth of CRISPR/Cas9-mediated knockout mutants *osinh2 #6*, *osinh2 #11*, *osinh3 #4*, and *osinh3 #9*, and overexpression lines *OsINH2-OE #1*, *OsINH2-OE #3*, *OsINH3-OE #1*, and *OsINH3-OE #4* in the greenhouse (Supplementary Figure 4). The phenotypic observation revealed that the seedling growth of *OsINH2* and *OsINH3* lines was similar to their corresponding wild type (ZH11) (Supplementary Figure 4). However, at the reproductive stage, *OsINH2* and *OsINH3* knockout lines were less fertile, while overexpression lines showed the same fertility as ZH11 (Figures 3A,E). The number of seeds reduced significantly in *osinh2 #6*, *osinh2 #11*, *osinh3 #4*, and *osinh3 #9* lines. Whereas, in overexpression lines, the number of seeds was not markedly different from ZH11 (Figures 3B,F). Therefore, it was exciting to find out whether the *OsINH2* and *OsINH3* affect the rice yield by affecting pollen grains. Surprisingly, the anthers in knockout lines of *OsINH2* and *OsINH3* were dried, orange brown (Supplementary Figure 4), and possessed less active pollen grains in comparison with ZH11 (Figures 3C,G). The percentage of active pollen was significantly lower in knockout lines of OsINH2 and OsINH3, while the overexpression lines possessed the same pollen viability as the ZH11 (Figures 3D,H). Thus, it indicates that *OsINH2* and *OsINH3* might play a crucial role in the pollen development of rice.

### *OsINH2* and *OsINH3* participated in ABA stress response

**Figure 4:**
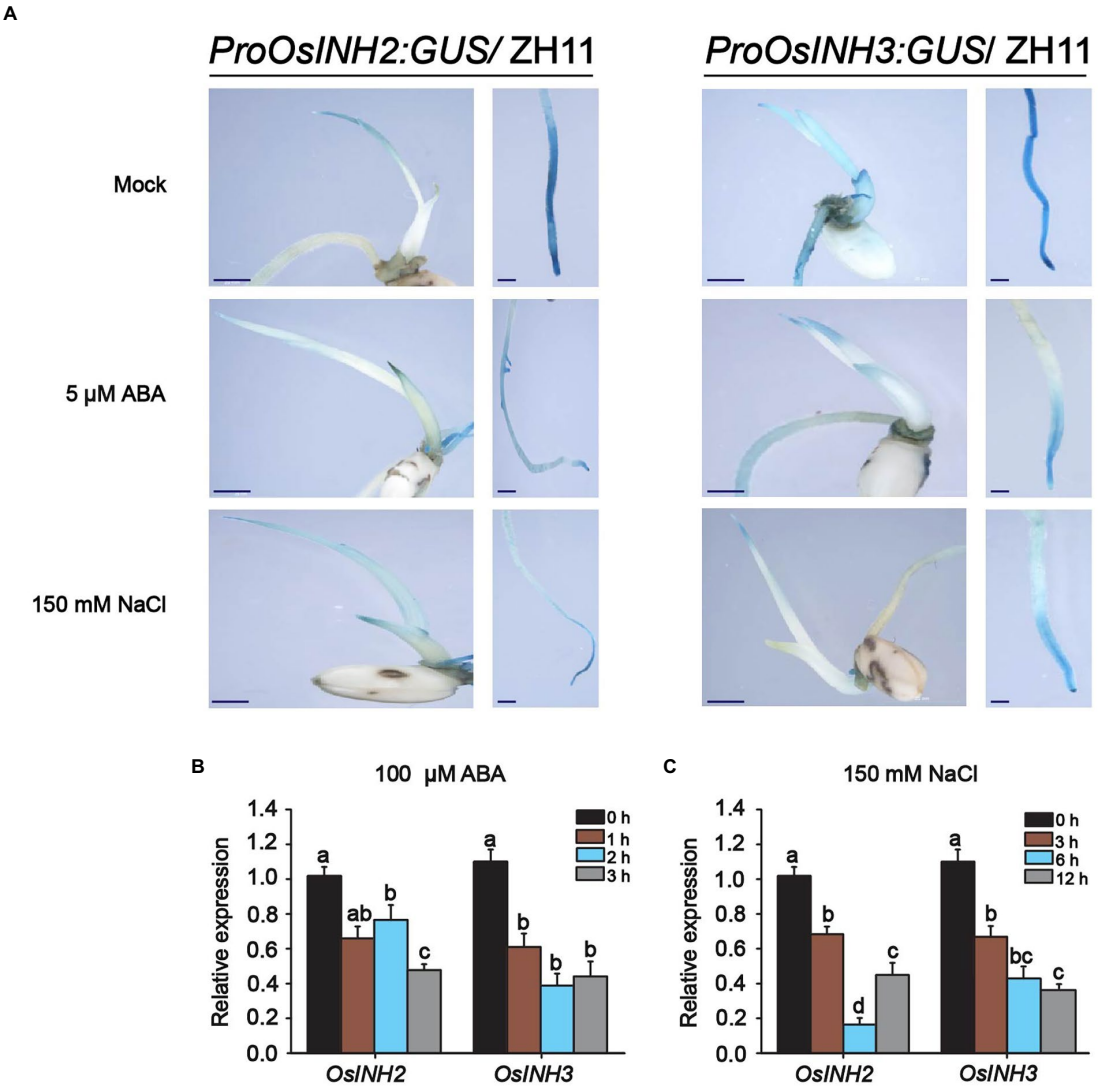
Expression patterns of *OsINH2* and OsINH3 under ABA and NaCl. **(A)** GUS activity of *OsINH2* and *OsINH3* were observed by GUS staining at the germination stage. Seeds were germinated on ½ MS media without and with 150 mM NaCl and 5 μM ABA. Scale bars, 20 mm. **(B,C)** Expression levels of *OsINH2* and *OsINH3* under 100 μM ABA and 150 mM NaCl stress. Seeds were germinated on ½ MS media and one-week-old seedlings were transferred to the nutrient solution. At the three-leaf stage, leaves were sprayed with 100 μM ABA for expression levels under ABA and sampled at 0, 1, 2, and 3 h. For expression under NaCl treatment, seedlings were transferred to nutrient solution supplemented with 150 mM NaCl and leaves were sampled at 0, 3, 6, and 12 h. Total RNA was extracted from leaves. Graph bars represent mean data. Error bars show ±SE with biological triplicates. The *UBQ10* was used as a reference gene. Different letters on the graph bars indicate statistical significant differences analyzed by one-way ANOVA at *p* < 0.05.

**Figure 5:**
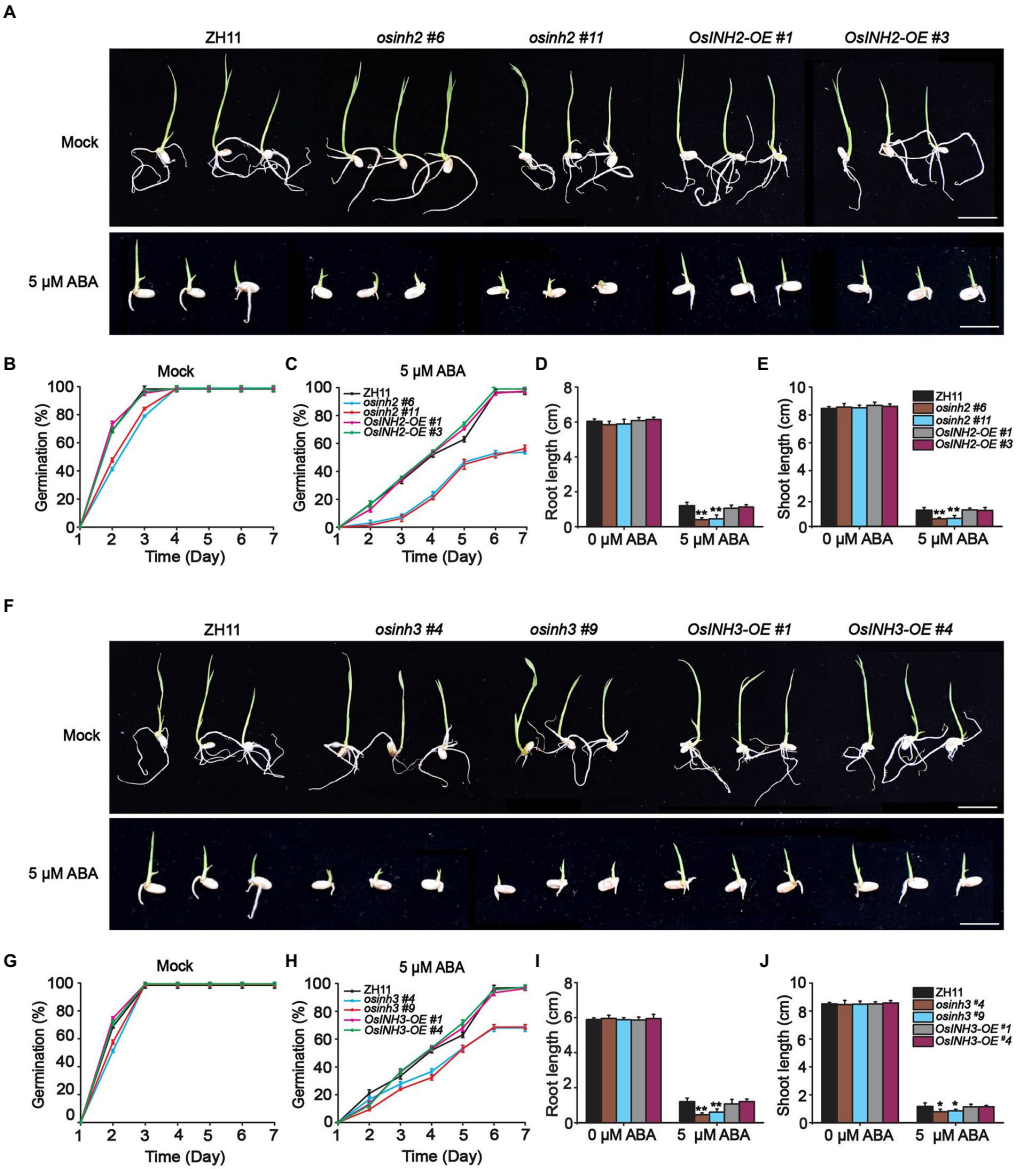
*osinh2* and *osinh3* mutants were sensitive to ABA. **(A)** Phenotypes of *OsINH2* knockout and overexpression lines at germination stage grown in media without and with ABA. Scale bars, 2 cm. **(B,C)** Graphical illustration of *OsINH2* seed germination (%) under mock and ABA treatment. **(D,E)** Indicating root and shoot length of *OsINH2* seedlings. **(F)** Phenotypes of *OsINH3* genotypes at germination stage in media without and with ABA. Scale bars, 2 cm. **(G,H)** Graphical illustration of *OsINH3* seed germination (%) under mock and ABA treatment. **(I,J)** Represents root and shoot length of *OsINH3* seedlings. Seeds were germinated on ½ MS media supplemented with 0 and 5 μM ABA. Germination (%) was recorded for up to 7 days. In **(B,C,G,H)** graph lines represent mean data. Error bars in **(B–J)** show ±SE with biological triplicates (*n* = 20). Seed germination (%) was analyzed by one-way ANOVA, followed by the Tukey’s test and significance was calculated at *p* < 0.05. Root and shoot length were analyzed by student’s *t*-test. Asterisks in **(D,E,I,J)** specify the significant difference between the ZH11 and transgenic lines, ^*^p < 0.05; ^**^p < 0.01.

Abscisic acid biosynthesis under various stress conditions, the role of phosphoproteins in complex signaling networks (Yin et al., 2018), and the negative regulation of ABA signaling by the INH2-TOPPs complex (Hou et al., 2016), incited us to study the involvement of *OsINH2* and *OsINH3* in rice under exogenous ABA. To clarify their role under stress conditions, firstly we checked their expression profiles precisely under ABA and NaCl stress. The *GUS* activity at the germination stage demonstrated that both ABA and NaCl applications reduced the expression of *OsINH2* and *OsINH3* (Figure 4A). Similarly, the qRT-PCR analysis also confirmed that the transcription levels of *OsINH2* and *OsINH3* were remarkably downregulated under ABA and NaCl (Figures 4B,C), which proposed that these proteins may play a fundamental role in ABA and NaCl signaling transduction. Thus, we observed their involvement in rice growth under ABA. The seed germination of *OsINH2* and *OsINH3* knockout and overexpression lines was the same as ZH11 in normal conditions. However, under ABA treatment, knockout lines showed ABA-mediated repression of seed germination (Figures 5A,F). The *osinh2 #6*, *osinh2 #11*, *osinh3 #4*, and *osinh3 #9* lines showed a remarkable reduction in seed germination percentage (Figures 5B,C,G,H). Moreover, besides seed germination, ABA also retarded seedling growth of *osinh2* and *osinh3* lines as their root and shoot growth reduced markedly in comparison with ZH11 (Figures 5D,E,I,J). Although seed germination and seedling growth of *OsINH2* and *OsINH3* overexpression lines were similar to ZH11, they showed resistance to ABA stress as compared to their respective knockout lines (Figure 5). In addition, the relative germination percentage at 4 DAG demonstrated that it was remarkably reduced in all the knockout lines, while overexpression lines possessed the same relative germination as ZH11 (Supplementary Figure 7). Therefore, our results endorse that *OsINH2* and *OsINH3* participate in ABA-mediated inhibition of seed germination and seedling growth of rice.

### OsINH2 and OsINH3 interacted with OsSAPKs

**Figure 6:**
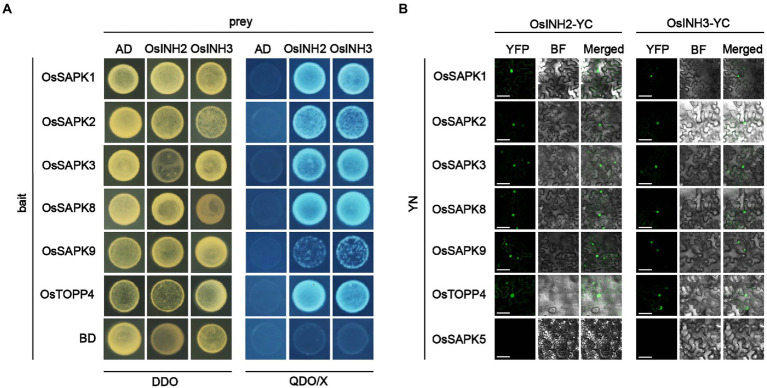
OsINH2 and OsINH3 interacted with OsSAPKs. **(A)** Y2H represents the interaction of OsINH2 and OsINH3 with OsSAPKs. OsINH2-AD and OsTOPP4-BD or OsINH3-AD and OsSAPK4-BD were used as positive control. OsINH2 and OsINH3 fused with empty AD and OsSAPKs fused with empty BD were used as negative control. DDO, SD/−Leu/−Trp; QDO/X, SD/−Leu/−Trp/-His/−Ade supplemented with X-α-Gal. **(B)** BiFC assay shows OsINH2-YC and OsINH3-YC interacted with OsSAPKs. OsINH2-YC and OsSAPKs-YN or OsINH3-YC and OsSAPKs-YN were co-expressed in *Nicotiana benthamiana* leaves. OsINH2-YC and OsTOPP4-YN or OsINH3-YC and OsTOPP4-YN were used as positive control. OsINH2-YC and OsSAPK5-YN or OsINH3-YC and OsSAPK5-YN were used as negative control. BF, bright field. Scale bars, 50 μm.

Previously, it is determined that mammal’s inhibitor-2 recruits various substrates (Brautigan, 2013), and the AtINH2-SnRKs complex negatively regulates ABA signaling in Arabidopsis (Hou et al., 2016). Thus, we hypothesized that in rice OsINH2 and OsINH3 may mediate cellular responses under ABA by targeting kinases (OsSAPKs). As anticipated, in the Y2H system, OsINH2 and OsINH3 linked to the Gal4 activation domain interacted with some of the OsSAPKs fused to the DNA-Gal4 binding domain. Similar to their alike responses in growth and ABA stress, their substrate recruitment was also alike, as both regulatory proteins showed interaction with the same OsSAPKs including OsSAPK1, OsSAPK2, OsSAPK3, OsSAPK8, and OsSAPK9 (Figure 6A). We then further verified their interaction *in vivo* by BiFC assay. As expected, co-expression of OsINH2-YFP^c^, and OsSAPK-YFP^n^ showed strong fluorescence signals in the nucleus and weak signals were reported in the cytoplasm. However, OsINH3-YFP^c^ interacted with OsSAPKs-YFP^n^ mainly in the nucleus (Figure 6B). Taken together, our findings suggest that OsINH2 and OsINH3 might involve in ABA signaling transduction through OsSAPKs.

### *OsINH2* and *OsINH3* played positive role in osmotic adjustment

**Figure 7:**
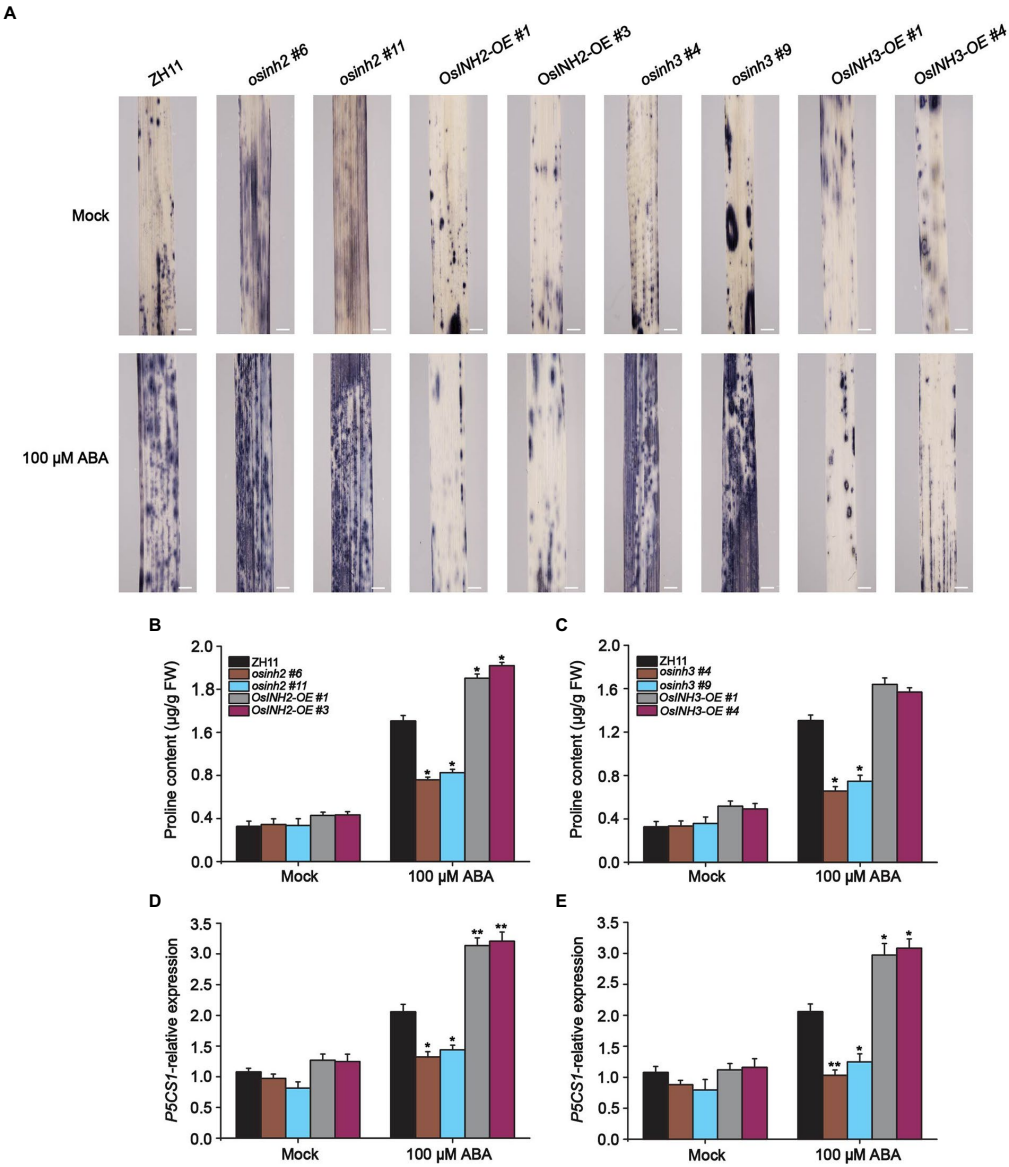
*OsINH2* and *OsINH3* positively regulated osmotic adjustment. **(A)** ROS accumulation in *OsINH2* and *OsINH3* genotypes. Seeds were germinated on ½ MS media. One-week-old seedlings were transferred to the nutrient solution and sprayed at the three-leaf stage with 100 μM ABA. Leaves samples were incubated at 37°C in NBT solution overnight and ROS were observed after decolorization with 75% ethanol. Scale bars, 10 mm. The experiment was repeated three times with the same results. **(B,C)** Proline content in *OsINH2* and *OsINH*3 lines. **(D,E)** Expression profile of *OsP5CS1* in *OsINH2* and *OsINH3* lines. Growth conditions and ABA application were similar as described in **(A)**. Proline content was assessed in leaves. In **(B–E)** graph bars represent mean data. Error bars show ±SE, with three biological repeats (*n* = 5 for proline content, *n* = 3 for *OsP5CS1* expression level). For all experiments, samples were collected before and after ABA treatment. Asterisks indicate a statistically significant difference between the ZH11 and transgenic lines was analyzed by Student’s *t*-test, ^*^p < 0.05; ^**^p < 0.01.

Recently Chen et al. (2022) reported that ABA induces ROS which represses seed germination and induces osmotic stress. Thus, we observed ROS accumulation in all genotypes under normal conditions and ABA. The detached leaves were subjected to nitroblue tetrazolium (NBT) staining overnight, and after decolorization with 75% ethanol, observed under a light microscope. We found that leaves of all the knockout lines were stained darker, in contrast, leaves from overexpression lines were less dark than ZH11 (Figure 7A). This prompted us to determine the contribution of *OsINH2* and *OsINH3* in osmotic adjustment *via* osmolyte production. The proline, a compatible osmolyte serves as a stress indicator influencing adaptive responses, by stabilizing subcellular structures and facilitating cell recovery after stress damage (Zhang et al., 2011). As a result, under ABA, knockout lines of both *OsINH2* and *OsINH3* accumulated remarkably less free proline content, while in overexpression lines it enhanced significantly over ZH11 (Figures 7B,C). Moreover, the expression level of the proline biosynthesis gene, delta-1-pyrroline-5-carboxylate synthase 1-like (*OsP5CS1*), significantly decreased in *osinh2* and *osinh3* lines, whereas overexpression lines showed a noticeable induction of *OsP5CS1* after ABA treatment (Figures 7D,E), which confirmed our above-mentioned results. These findings recommend that *OsINH2* and *OsINH3* might protect rice by balancing osmotic status, thereby minimizing ROS imbalance.

### *OsINH2* and *OsINH3* were involved in ROS detoxification

**Figure 8:**
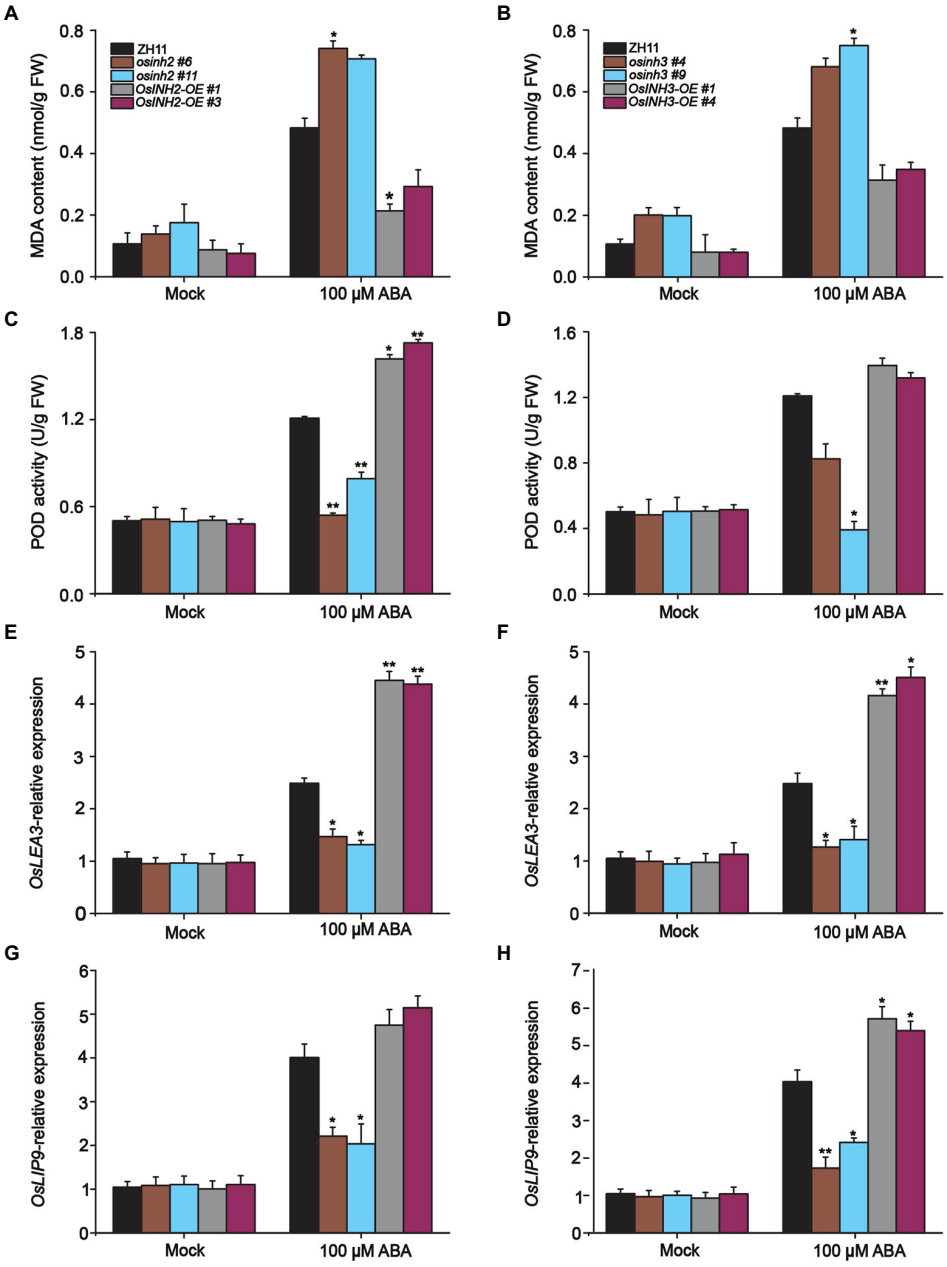
Participation of *OsINH2* and *OsINH3* in ROS detoxification. **(A,B)** MDA content in *OsINH2* and *OsINH3* lines. **(C,D)** POD activity in *OsINH2* and *OsINH3* genotypes. Seeds were germinated on ½ MS media. One-week-old seedlings were transferred to nutrient solution and at the three-leaf stage sprayed with 100 μM ABA. MDA content and POD activity were detected from leaves. **(E–H)** Expression profile of *OsLEA3* and *OsLIP19* in *OsINH2* and *OsINH3* lines. Growth conditions and ABA application were the same as mentioned above. Graph bars indicate mean data. Error bars show ±SE with three biological repeats (*n* = 5 for MDA and POD activity, for *OsLEA3* and *OsLIP19* expression levels, *n* = 3). Asterisks represent a statistically significant difference between the ZH11 and transgenic lines was analyzed by Student’s *t*-test, ^*^p < 0.05; ^**^p < 0.01.

It is reported that ABA induces ROS accumulation and causes oxidative stress in leaves which led to membrane damage (Hu et al., 2006). Thus, we predicted, along with ROS production, ABA also enhances the formation of malondialdehyde (MDA) content. Predictably, the MDA formation increased greatly in knockout lines among which *osinh2 #6*, and *osinh3 #9* showed significant MDA induction after ABA treatment. Whereas, overexpression lines showed reduction in MDA content, remarkably in *INH2-OE #1*, as compared to control (Figures 8A,B). These results proposed that under ABA stress *OsINH2* and *OsINH3* could protect against membrane damage by lowering lipid peroxidation formation.

Upon ROS imbalance and MDA formation, plants evolve numerous antioxidative enzymes to scavenge ROS (Blokhina and Fagerstedt, 2010). We speculated that *OsINH2* and *OsINH3* involve in tolerance mechanisms by antioxidant enzyme production. We analyzed the production of POD, CAT, and SOD in rice leaves before and after ABA treatment. Expectedly, POD was highly active to lower the ROS. The POD activity was noticeable in *OsINH2* knockout and overexpressing plants after ABA treatment. It was lower in *osinh2 #6* and *osinh2 #11* and higher in *INH2-OE #1* and *INH2-OE #3* than ZH11 (Figure 8C). While, in knockout and overexpression genotypes of *OsINH3*, except *osinh3 #9*, the POD activity was not significantly varied over ZH11 (Figure 8D). However, in all plants, neither CAT nor SOD was active under ABA. This suggests that *OsINH2* and *OsINH3* could regulate POD more actively than CAT and SOD and maintain ROS homeostasis.

Besides, we measured various stress-responsive genes which could either regulate the formation of protective contents or directly target other genes to confer tolerance against stressful conditions. The expression profile of well-known stress-responsive genes including *OsP5CS1*, NAC-domain containing protein 1(*NAC1*), late embryogenesis abundant 3 (*OsLEA3*), and ocs element-binding factor (*OsLIP9*) was assessed in respective plant materials. Presumably, in response to ABA, all knockout lines showed significant downregulation of *OsP5CS1*, *OsLEA3*, and *OsLIP9* gene expression in comparison with control (Figures 8E–H). However, the *OsNAC1* transcription was also reduced but not significantly (Supplementary Figure 8). In contrast, ABA induced the transcriptomic profile of these genes in overexpression genotypes (Figures 7E–H). These findings propose that *OsINH2* and *OsINH3* might contribute to ROS fine-tuning by stimulating antioxidant enzyme production and triggering stress-responsive genes that could play role in stress adaptation directly or indirectly.

### OsINH2*–*OsINH3 formed complex

**Figure 9:**
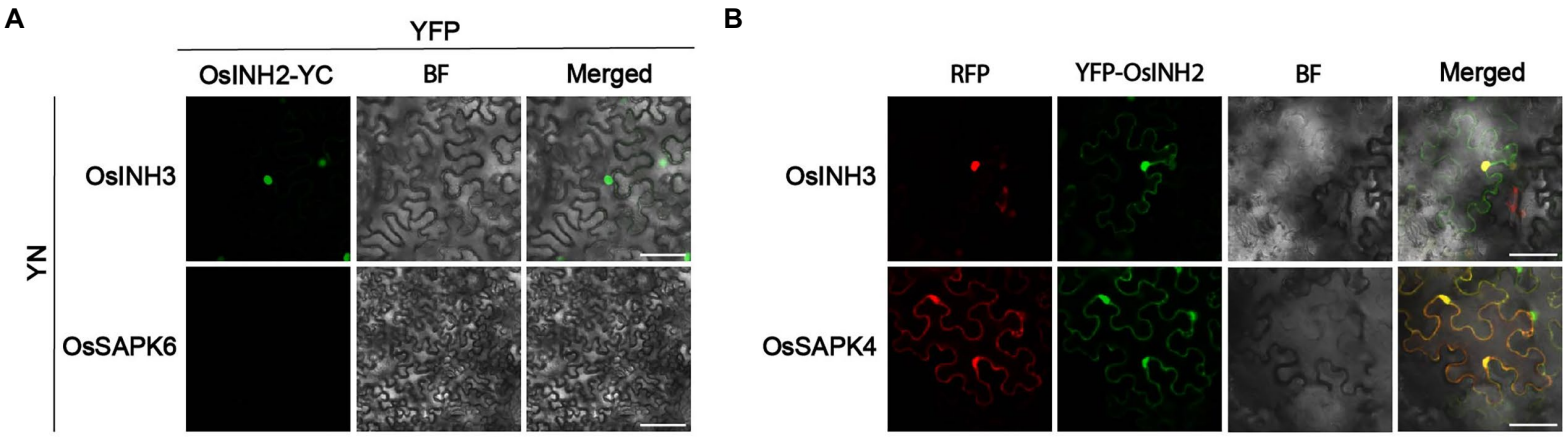
OsINH2 and OsINH3 interacted with each other. **(A)** BiFC assay showing the interaction of OsINH2 with OsINH3 *in vivo*. OsINH2-YC and INH3-YN were co-expressed in *N. benthamiana* leaves. Scale bars, 50 μm. OsINH2-YC and OsSAPK6-YN were used as negative control. **(B)** Transient expression assay shows YFP-OsINH2 colocalized with OsINH3-RFP. YFP-OsINH2 and OsSAPK4-RFP were used as positive control. All samples were injected into *Nicotiana benthamiana* leaves Scale bars, 25 μm.

As we reported in the above-mentioned results, the characterization, growth, and adaptive responses of *OsINH2* and *OsINH3* were alike. So, we hypothesized that OsINH2 and OsINH3 form complex and regulate different functions in the plants. We co-expressed OsINH2-YFP^c^ with OsINH3-YFP^n^ in *N.benthamiana* leaves. Surprisingly, strong fluorescence signals were observed in the nucleus (Figure 9A), which revealed that these two regulatory proteins in rice form a big complex and contribute decisively to rice growth. Moreover, YFP-OsINH2 colocalized with OsINH3-RFP in tobacco leaves and their subcellular localization remained unchanged (Figure 9B). Our findings elucidate that the OsINH2-OsINH3 complex may recruit other substrates and supervise various activities in the plants collectively or in co-regulation.

## Discussion

Protein phosphatases are strongly conserved enzymes that encode large gene families with versatile cellular progressions. To date, research on PP1 has mostly focused on the role of TOPPs (PP1c) in plants. However, the PP1r especially in crop growth and stress responses has not gained much attention. Thus, little knowledge of PP1r has attracted our attention to explore the contribution of *OsINH2* and *OsINH3* in rice. The expression features and localization demonstrated that these inhibitor proteins may supervise cellular processes all over the plant and at various locations in the cell, which explain their functional redundancy. Moreover, the interaction of OsINH2 and OsINH3 with OsTOPPs confirmed they recruit OsTOPPs. However, the OsTOPPs interaction with OsINH2^V6A/W8A^ and OsINH3^V50A/W52A^, having mutation in RVXF motif has revealed that rather than RVXF, there might be other potential motifs *via* which rice PP1r interacts with their complementary subunits and substrates. For instance, Templeton et al. (2011) reported two other conserved motifs in Inhibitor-2 (I-2), the PXTP, and HYNE. Further, OsINH2 and OsINH3 colocalization with OsTOPPs ensured they are counter partners of PP1c in rice and might define their activities without changing their location in the cell. In contrast, in Arabidopsis, INH3-W43A diminished the TOPP’s nuclear localization and PRSL1 targeted PP1c to the cytoplasm (Takemiya et al., 2009, 2013). The co-localization of OsINH2 with OsTOPPs was similar to the colocalization of PP1R3 with TOPP4 (Zhang et al., 2020).

Growth observation revealed that *OsINH2* and *OsINH3* were involved in the reproductive growth of rice, as a mutation in *OsINH2* and *OsINH3* affected rice fertility. Therefore, the pollen inactivity of the knockout lines explains that reduced pollen grains viability may lead to fewer seeds. Moreover, after ABA application, premature spikelet abortion and a remarkable decrease in the number of seeds (Oliver et al., 2007) provide a clue that *OsINH2* and *OsINH3* might be involved in fertility by interrupting ABA signaling. Furthermore, the Low Seed Setting Rate 1 (*OsLSSR1*), which regulates the seed setting rate in rice (Xiang et al., 2019) might be affected in knockout lines as a result seed number became decreased. Besides, embryogenesis regulation by *AtINH3* (Takemiya et al., 2009) and short fluorescence and less fertility of Arabidopsis *topp4-1* double mutant (Qin et al., 2014) also confirmed that PP1 regulates yield-related traits. Additionally, the expression of *OsINH2* and *OsINH3* in the panicles and flowers further supported our notion that the regulatory proteins may contribute to the reproductive growth of rice. Besides, we found all the five isoforms of OsTOPP single knockout mutants have not shown obvious phenotype at the reproductive stage, which might be due to their functional redundancy. However, the *ostopp1-2* double mutant showed the same fertility phenotype as *osinh2* and *osinh3* lines (data not published). Moreover, the role of SAPK10 in rice flowering (Liu et al., 2019) and SAPK2 in rice yield under reproductive stage drought stress (Lou et al., 2020) demonstrated that OsSAPKs might contribute to rice reproductive growth along with OsINH2 and OsINH3, which could be explained clearly by observing their cross material. While the similar response of overexpression lines to ZH11 demonstrated that *OsINH2* and *OsINH3* active involvement in growth may depend on their specific expression level and interaction with other yield regulating genes, which could suppress their activity.

Given that OsINH2 and OsINH3 interacted with OsTOPPs, we investigated whether, like TOPPs, they also contribute to stress signaling. The disruption of *OsINH2* and *OsINH3* under ABA treatment has delayed the seed germination and impaired seedling growth in knockout lines in comparison with ZH11. These results indicated that mutation of *OsINH2* and *OsINH3* causes inhibition of rice growth under ABA which suggests their involvement in growth responses might be *via* ABA-dependent complex signaling. However, the response of overexpression lines explains that too high or too low expression of *OsINH2* and *OsINH3* may affect their performance in some growth responses. Our results are consistent with the involvement of *PP1R3* in stress responses, as Zhang et al. (2020) reported a mutation in *PP1R3* not only results in decreased seed germination and cotyledon greening but also retards seedling growth under ABA. Moreover, under exogenous brassinosteroid (BR), longer roots of *TdPP1* overexpressing transgenic lines (Bradai et al., 2021) confirmed the participation of PP1 under hormonal stress in crops. Similarly, NaCl tolerance of *OsPP1a* overexpression genotypes (Liao et al., 2016) highlighted that PP1 could also rescue plants under abiotic stresses. Besides, their interaction with the ABA signaling components (OsSAPKs) stated that they might mediate cellular responses under stress by ABA-dependent pathways, similar to the mechanism in Arabidopsis. Based on previous findings that Os*SAPK1* and Os*SAPK2* positively regulate salt tolerance in rice (Lou et al., 2018), we suggested that *OsINH2* and *OsINH3* may play their potential role in stress adaptations by targeting OsSAPKs.

Recently, Chen et al. (2022) have reported that ROS generation by ABA causes inhibition of seed germination, which recommended that mutation in OsINH2 and OsINH3 may inhibit seed germination through ROS, thus we investigated ROS accumulation. ROS is a secondary messenger which mediates different responses in the cell being disrupted by various stressors. In the present research, under ABA more ROS accumulation in knockout lines, while less ROS in overexpression lines illustrated that *OsINH2* and *OsINH3* could play a positive role in osmotic adjustment and overcome ROS toxicity. Similarly, Shi et al. (2012) reported oxidative damage in rice under ABA stress. Further, Liu et al. (2020) studied ROS burst is enhanced in the *topp4-1* mutant in Arabidopsis, which signified the contribution of PP1 in ROS signaling. Therefore, to deal with osmotic stress, plants have evolved the production of compatible solute (proline) and antioxidant defense systems (Goggin and Colmer, 2007; Li and Wang, 2013). The proline adjusts osmotic disturbance, scavenge ROS and stabilizes proteins and cell membrane (Székely et al., 2008; Bailey-Serres et al., 2012), while the antioxidant defense mechanisms enhance plant tolerance by ROS detoxification (Zaefyzadeh et al., 2009). In the current research, regulation of the proline content and proline biosynthesis gene (*OsP5CS1*) in all genotypes, clarified that *OsINH2* and *OsINH3* might adjust osmotic disruption *via* osmotically active metabolites. Likewise, Cao et al. (2020) and Li et al. (2021), reported different stressors including salinity, drought, ABA and osmotic disruption affect proline biosynthesis in various rice genotypes. In addition, MDA quantification verified more ROS generation induces MDA formation in knockout lines while less MDA content in overexpression lines referred that probably *OsINH2* and *OsINH3* protect rice *via* decreasing MDA content. Similarly, Liao et al. (2016) investigated lower MDA content in OsPP1a transgenic lines under salt stress and Djanaguiraman et al. (2010) also described excessive ROS generation results in increased MDA content. To sum up, OsINH2 and OsINH3 might rescue rice against membrane damage *via* proline production and MDA reduction. However, exploration of other possible pathways could deeply explain the role of *OsINH2* and *OsINH3* in ROS balancing and membrane protection.

Besides, an antioxidant enzyme, POD activity was noticeable in all genotypes in response to ABA which evidenced that, upon ROS imbalance, *OsINH2* and *OsINH3* might activate POD to disintegrate excessive H_2_O_2_ into water and molecular oxygen, and assist rice to adapt ABA elevation. The research findings that more nitric oxide (NO) generation enhance transcription and activities of antioxidative enzymes against ABA (Zhang et al., 2007) also supported our results. Likewise, Shu et al. (2016) stated exogenous ABA induces POD activity in pumpkins. Similarly, Ding et al. (2010) and Wang et al. (2013) verified that excessive ABA stimulates enzymatic as well as non-enzymatic protective strategies in different cold tolerant rice genotypes and common reed plants. Moreover, under ABA stress, *OsINH2* and *OsINH3* regulated the expression level of stress-responsive genes, such as *OsLEA3*, *OsLIP9,* and *OsNAC1* genes, which described that *OsINH2* and *OsINH3* might regulate adaptive strategies by peroxidases and coordinating with different stress-responsive genes which could activate various protective strategies. Finally, OsINH2 interaction and colocalization with OsINH3 pointed out that their similar behavior might be due to their co-regulation. Therefore, it is needed to underpin why these two inhibitor proteins, with different origins, sequences, and motifs, behave similarly and their contribution to different pathways.

**Figure 10:**
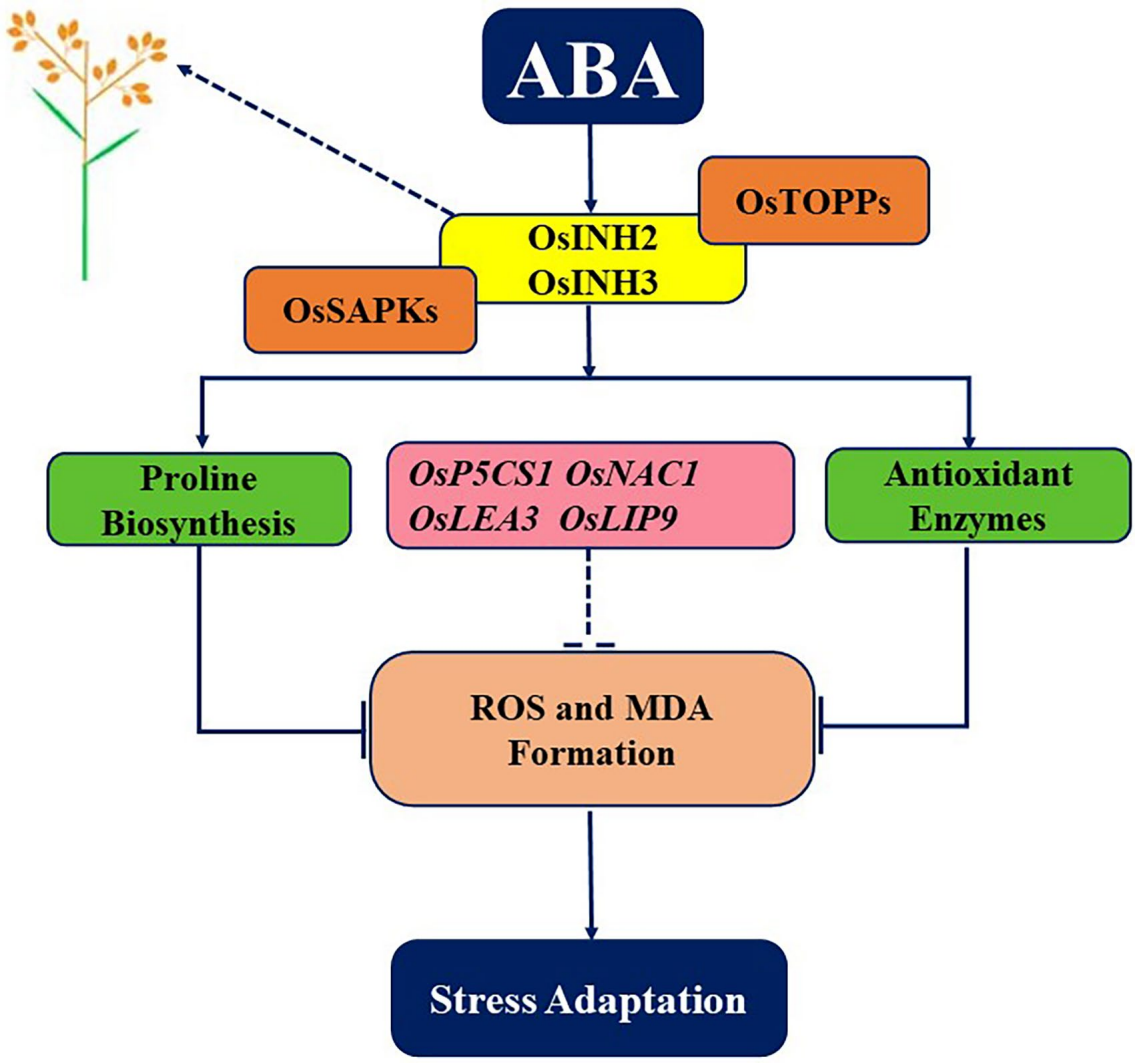
A graphical abstract depicting the role of *OsINH2* and *OsINH3* in rice. As regulatory subunits of PP1, OsINH2, and OsINH3 interact with OsTOPPs as well as with ABA signaling components (OsSAPKs). On one hand, they regulate the number of seeds in rice, and on other hand as a complex, they might regulate ABA-mediated repression of seed germination and maintain ROS levels by regulating antioxidants, osmolytes, and stress-responsive genes. Thereby, participating in the reproductive growth of rice and adaptative strategies under stress.

To conclude, our study provides key insights into the contribution of OsINH2 and OsINH3 to rice growth and positive responses under ABA. Current findings illustrate that OsINH2 and OsINH3 are ubiquitous complementary partners of OsTOPPs, which participate in reproductive growth and the ABA signaling cascade by interacting with OsSAPKs. They release ABA-mediated repression of seed germination and fine-tuned the adaptive strategy of rice under ABA stress by detoxifying ROS, promoting proline biosynthesis, inhibiting MDA formation, and regulating stress-responsive genes (Figure 10). However, *OsINH2* and *OsINH3* functioning may depend on their specific transcription level in different responses. In Arabidopsis, tissue-specific expression of INH2 and INH3, participation of INH3 in embryogenesis, and negative regulation of ABA signaling by INH2-TOPP-SnRKs complex (Takemiya et al., 2009; Hou et al., 2016), are the same as in rice. Our findings showed the involvement of ubiquitously expressed OsINH3 in rice reproductive growth, OsINH2 in rice seed germination under ABA, and interaction with ABA signaling components (SAPKs). This revealed that to some extent these two inhibitor proteins possessed functional conservation and similar expression features in rice and Arabidopsis. However, there is no knowledge about the contribution of INH2 to fertility and ROS adjustment in Arabidopsis. As well as the activity of AtINH3 under ABA *via* SAPKs is still elusive. Thus, the participation of INH2 in Arabidopsis reproductive growth, INH3 in ABA responses, and their involvement in ROS regulation could deeply explain the functional conservation of inhibitor proteins in Arabidopsis and crops. Our research explored for the first time the role of PP1r in crops and would contribute to introducing stress-resistant crops. However, further investigations are required to underpin underlying mechanisms widely.

## Data availability statement

The original contributions presented in the study are included in the article/Supplementary material, further inquiries can be directed to the corresponding author.

## Author contributions

SJ, QQ, and SH have designed the experiments. SJ has conducted the experimental work and data analyses and wrote the manuscript. SH and QQ have revised the manuscript. WS helped in plasmids construction. YL revised the manuscript and ensured equipment availability used in this research. All authors contributed to the article and approved the submitted version.

## Funding

We are grateful to National Natural Science Foundation of China, General Project, 31870251 and 32170340, the Fundamental Research Funds for the Central Universities (lzujbky-2021-45), Xinyu Wang for providing Y2H-related vectors, Quansheng Qiu for the pUBC-RFP-Dest vector, and the core faculty of School of Life Sciences, Lanzhou University.

## Conflict of interest

The authors declare that the research was conducted in the absence of any commercial or financial relationships that could be construed as a potential conflict of interest.

## Publisher’s note

All claims expressed in this article are solely those of the authors and do not necessarily represent those of their affiliated organizations, or those of the publisher, the editors and the reviewers. Any product that may be evaluated in this article, or claim that may be made by its manufacturer, is not guaranteed or endorsed by the publisher.

## Supplementary material

SUPPLEMENTARY FIGURE 1 Multiple sequence alignment INH2 and INH3 in rice and Arabidopsis. The sequences were aligned by using the ClustalW program. Asterisks represent an identical amino acid sequence. Dashes indicate gaps to allow for the optimal alignment of sequences. Redline indicates the RVXF motif (conserved region) among these proteins.

SUPPLEMENTARY FIGURE 2 Tissue-specific expression of *OsINH2* and *OsINH3*. Seeds were germinated on ½ MS media for a week and then one-week-old seedlings were transferred into a nutrient solution. Total RNA was extracted at 3 leaf stage from roots, leaves, and stems, while from florets and flowers after flowering. The expression level is indicated as a relative value, and expression in root was defined as 1.0. The graph bars represent mean data. The *UBQ10* was used as a reference gene. Error bars indicate ± SE. The expression level was analyzed by student’s *t* test at *p* < 0.05.

SUPPLEMENTARY FIGURE 3 OsINH2^V6A/W8A^ and OsINH3^V50A/W52A^ interacted with OsTOPPs. **(A)** Y2H shows the interaction of OsINH2^V6A/W8A^ and OsINH3^V50A/W52A^ with OsTOPPs. OsINH2^V6A/W8A^ -AD with OsSAPK1-BD or OsINH3^V50A/W52A^ -AD with OsSAPK1-BD were used as positive control. OsINH2^V6A/W8A^ and OsINH3^V50A/W52A^ fused with empty AD and OsTOPPs fused with empty BD were used as negative control. DDO, SD/-Leu/-Trp; QDO/X, SD/-Leu/-Trp/-His/-Ade supplemented with X-α-Gal. **(B)** BiFC assay represents the interaction of OsINH2^V6A/W8A^ and OsINH3^V50A/W52A^ with OsTOPPs *in vivo*. OsINH2^V6A/W8A^ -YC and OsINH3^V50A/W52A^ -YC were co-expressed with OsTOPPs-YN in *N. benthamiana* leaves. OsINH2^V6A/W8A^ -YC and OsSAPK1 or OsINH3^V50A/W52A^ -YC and OsSAPK1-YN were used as positive control. OsINH2^V6A/W8A^ -YC and OsSAPK5 or OsINH3^V50A/W52A^ -YC and OsSAPK5-YN were used as negative control.

SUPPLEMENTARY FIGURE 4 Identification of *OsINH2* and *OsINH3* genotypes. **(A,B)** Sequencing analysis of CRISPR/ Cas9-mediated *OsINH2* and *OsINH3* knockout lines. Red lines represent the insertion or deletion of nucleotides in homozygous mutants. **(C,D)** Relative expression of *OsINH2* and *OsINH3* in overexpression lines. Seeds were germinated on ½ MS media for a week and then seedlings were transferred to the nutrient solution. Total RNA was extracted from leaves at the three-leaf stage. Graph bars represent mean data. Error bars indicate ±SE with three biological repeats. The *UBQ10* was used as a reference gene The expression levels were analyzed by student’s *t*-test. Asterisks specify the significant difference between the ZH11 and overexpression lines, **p* < 0.05; ^**^*p* < 0.01.

SUPPLEMENTARY FIGURE 5 Growth of *OsINH2* and *OsINH3* lines at the seedling stage. **(A,B)** Growth of *OsINH2* and *OsINH3* knockout and overexpression lines. Seedlings were grown on ½ MS media for 14^th^ days and photographed. Scale bars, 2 cm.

SUPPLEMENTARY FIGURE 6 Microscopic observation of flowers and anthers of *OsINH2* and *OsINH3* genotypes. **(A)** Flower phenotype of *OsINH2* lines. **(B)** Stained anthers of *OsINH2* genotypes. **(C)** Flower phenotype of *OsINH3* lines. **(D)** Stained anthers of *OsINH3* genotypes. Flowers enclosed in bracts were selected for microscopic observation. Anthers were stained by 1–2 drops of potassium iodide solution (KI). In **(B,D)** anthers represent dark blue (active) enclosed pollens, scale bars, 20 mm.

SUPPLEMENTARY FIGURE 7 Relative germination percentage of *OsINH2* and *OsINH3*. **(A,B)** Relative germination percentage of *OsINH2* and *OsINH3* lines. Seeds were germinated on ½ MS media with and without ABA. The germination (%) was recorded on the 4th day. The experiment was repeated three times with similar results.

SUPPLEMENTARY FIGURE 8 Relative expression of *OsNAC1* in *OsINH2* and *OsINH3* lines under ABA treatment. **(A,B)** Expression profile of *OsNAC1* in *OsINH2* and *OsINH3* transgenic materials. Seeds were germinated on ½ MS media and one-week-old seedlings were transferred to nutrient solution and sprayed with 100 μM ABA at the three-leaf stage. Total RNA was extracted from leaves. Graph bars represent mean data. Error bars show ± SE in three repeats. The *UBQ10* was used as a reference gene. The expression level was analyzed by student’s *t*-test at *p* < 0.05.
