# Supplementary figures and images for: Rice protein phosphatase 1 regulatory subunits OsINH2 and OsINH3 participate actively in growth and adaptive responses under abscisic acid
Source: Front Plant Sci. 2022 Sep 7;13:990575. doi: 10.3389/fpls.2022.990575 (PMC9521630; doi:10.3389/fpls.2022.990575)

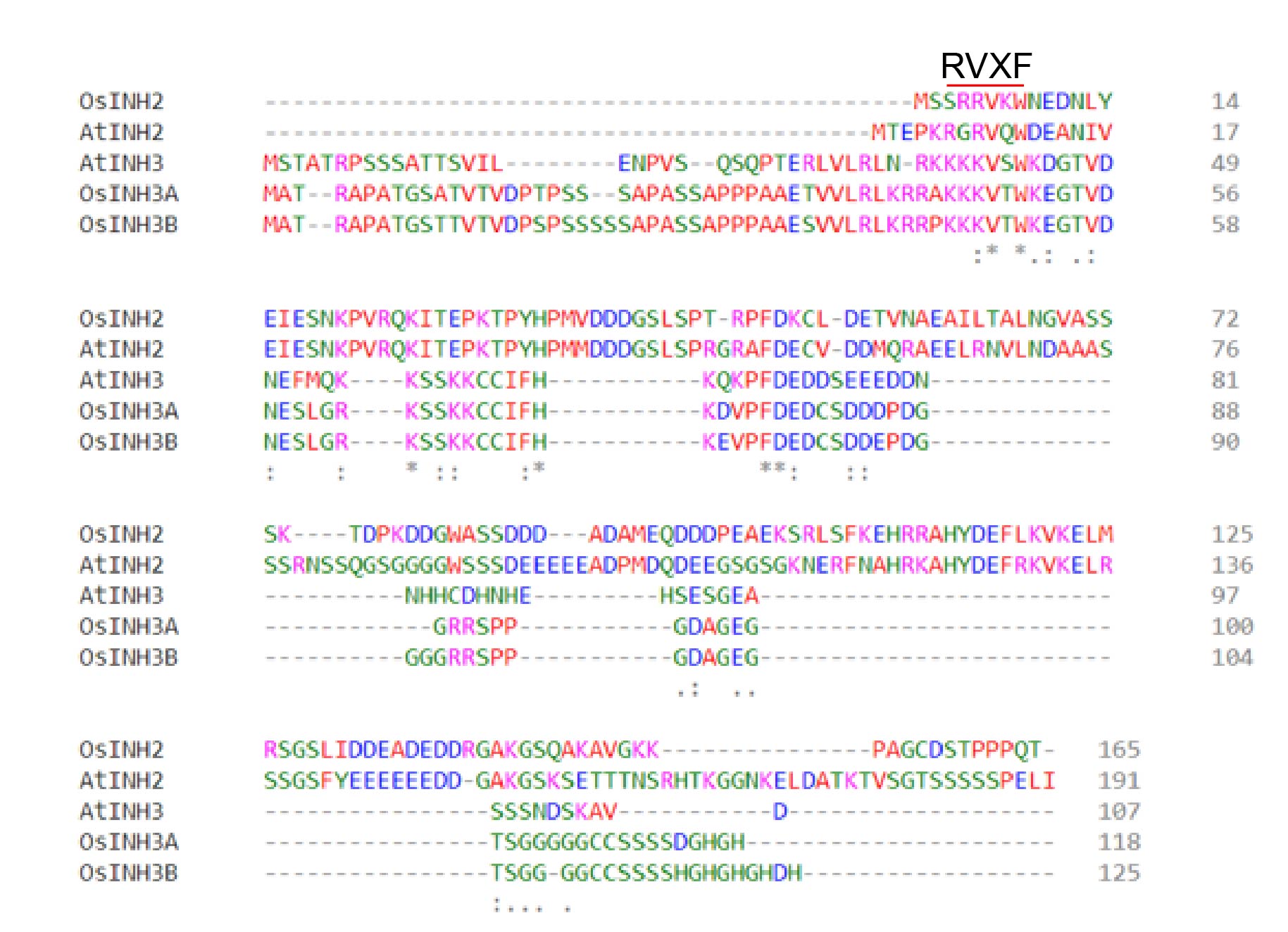

Supplement: SUPPLEMENTARY FIGURE 1 — Multiple sequence alignment INH2 and INH3 in rice and Arabidopsis. The sequences were aligned by using the ClustalW program. Asterisks represent an identical amino acid sequence. Dashes indicate gaps to allow for the optimal alignment of sequences. Redline indicates the RVXF motif (conserved region) among these proteins. [file Image_1.JPEG]

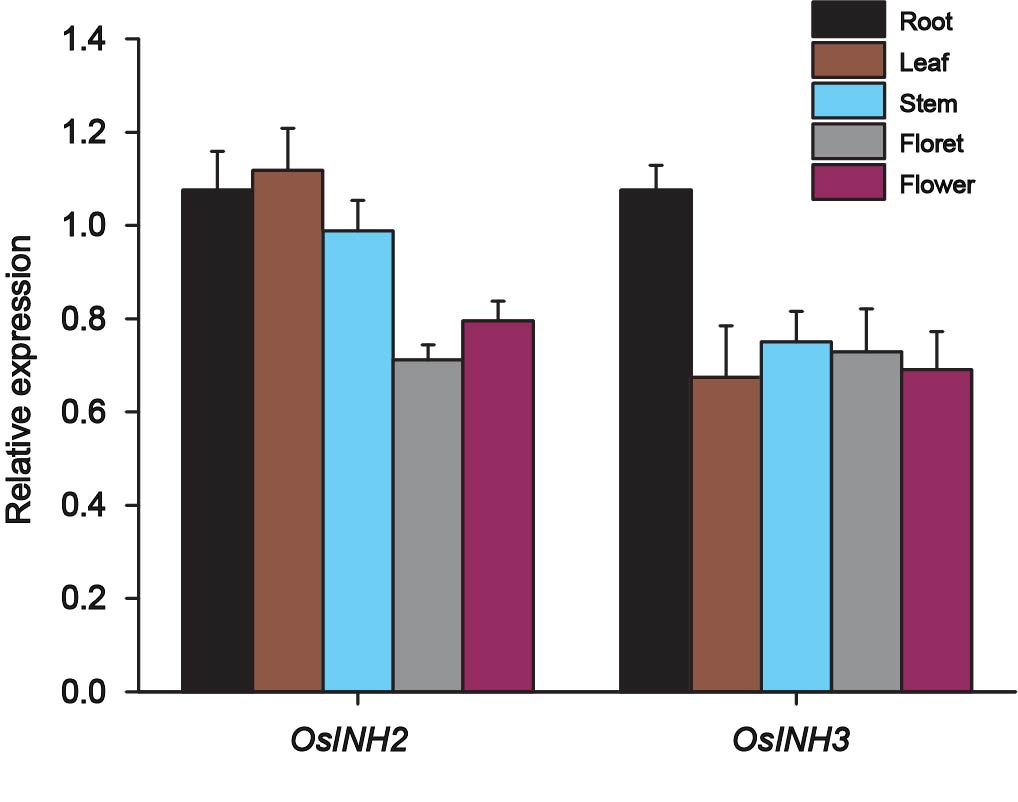

Supplement: SUPPLEMENTARY FIGURE 2 — Tissue-specific expression of OsINH2 and OsINH3. Seeds were germinated on ½ MS media for a week and then one-week-old seedlings were transferred into a nutrient solution. Total RNA was extracted at 3 leaf stage from roots, leaves, and stems, while from florets and flowers after flowering. The expression level is indicated as a relative value, and expression in root was defined as 1.0. The graph bars represent mean data. The UBQ10 was used as a reference gene. Error bars indicate ± SE. The expression level was analyzed by student’s t test at p < 0.05. [file Image_2.JPEG]

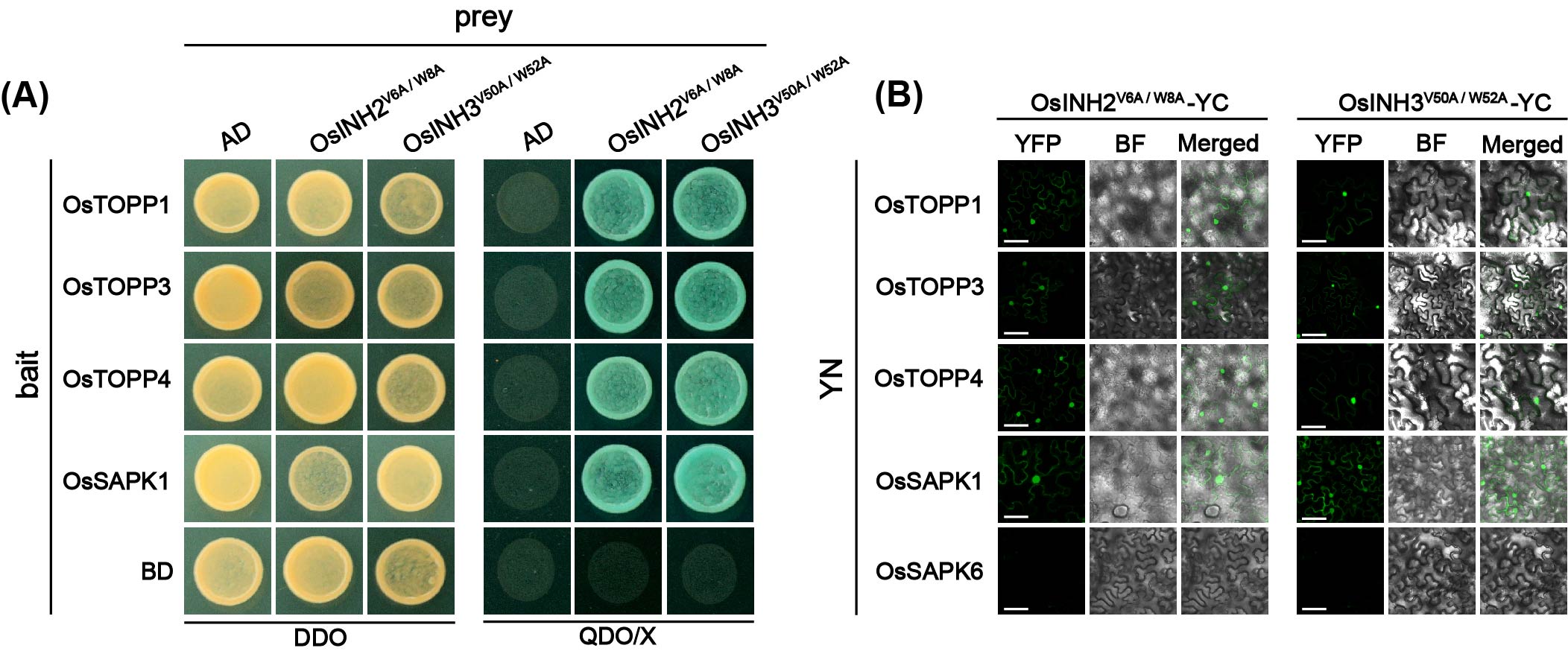

Supplement: SUPPLEMENTARY FIGURE 3 — OsINH2V6A/W8A and OsINH3V50A/W52A interacted with OsTOPPs. (A) Y2H shows the interaction of OsINH2V6A/W8A and OsINH3V50A/W52A with OsTOPPs. OsINH2V6A/W8A -AD with OsSAPK1-BD or OsINH3V50A/W52A -AD with OsSAPK1-BD were used as positive control. OsINH2V6A/W8A and OsINH3V50A/W52A fused with empty AD and OsTOPPs fused with empty BD were used as negative control. DDO, SD/-Leu/-Trp; QDO/X, SD/-Leu/-Trp/-His/-Ade supplemented with X-α-Gal. (B) BiFC assay represents the interaction of OsINH2V6A/W8A and OsINH3V50A/W52A with OsTOPPs in vivo. OsINH2V6A/W8A -YC and OsINH3V50A/W52A -YC were co-expressed with OsTOPPs-YN in N. benthamiana leaves. OsINH2V6A/W8A -YC and OsSAPK1 or OsINH3V50A/W52A -YC and OsSAPK1-YN were used as positive control. OsINH2V6A/W8A -YC and OsSAPK5 or OsINH3V50A/W52A -YC and OsSAPK5-YN were used as negative control. [file Image_3.JPEG]

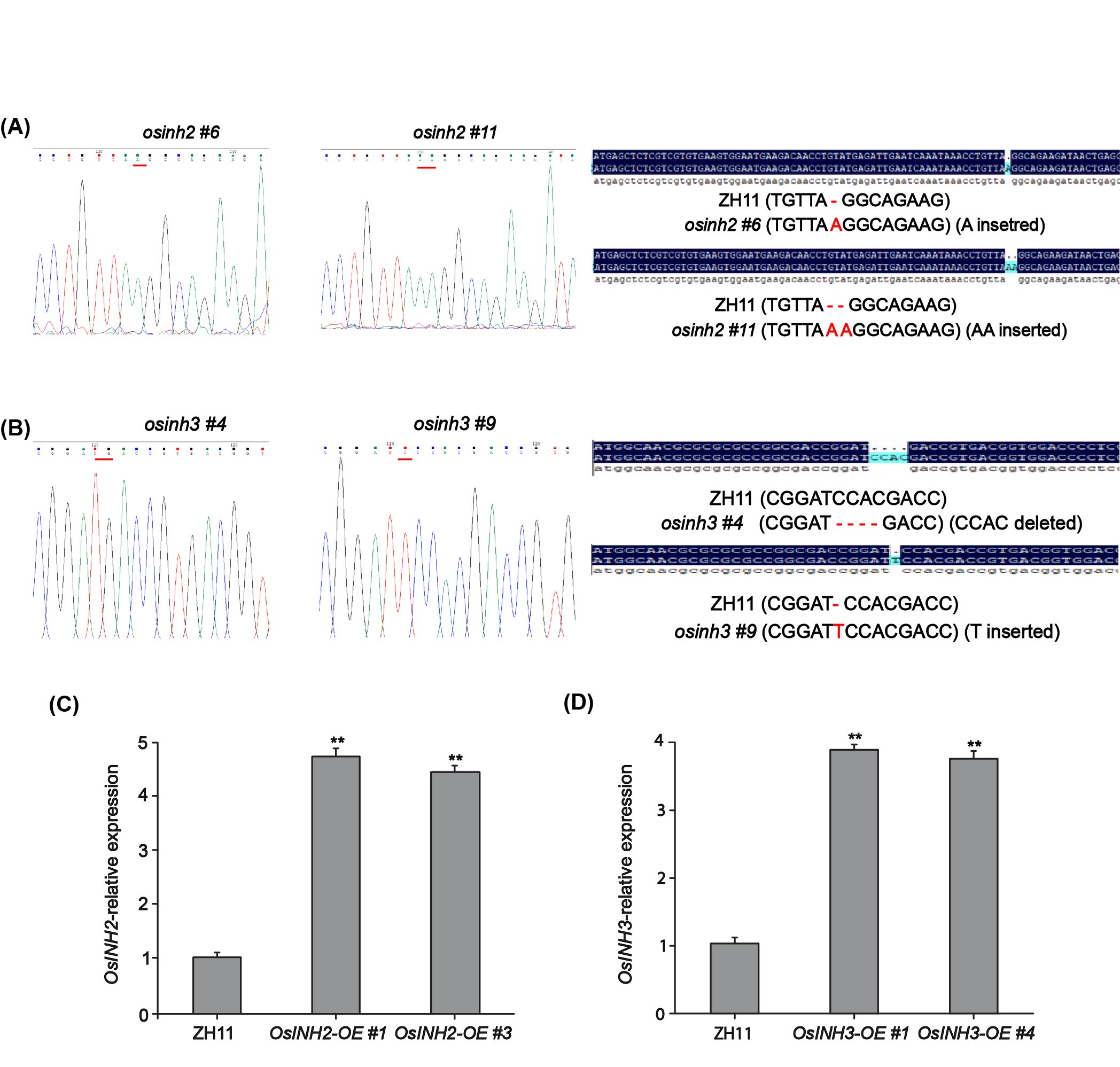

Supplement: SUPPLEMENTARY FIGURE 4 — Identification of OsINH2 and OsINH3 genotypes. (A,B) Sequencing analysis of CRISPR/ Cas9-mediated OsINH2 and OsINH3 knockout lines. Red lines represent the insertion or deletion of nucleotides in homozygous mutants. (C,D) Relative expression of OsINH2 and OsINH3 in overexpression lines. Seeds were germinated on ½ MS media for a week and then seedlings were transferred to the nutrient solution. Total RNA was extracted from leaves at the three-leaf stage. Graph bars represent mean data. Error bars indicate ±SE with three biological repeats. The UBQ10 was used as a reference gene The expression levels were analyzed by student’s t-test. Asterisks specify the significant difference between the ZH11 and overexpression lines, *p < 0.05; **p < 0.01. [file Image_4.JPEG]

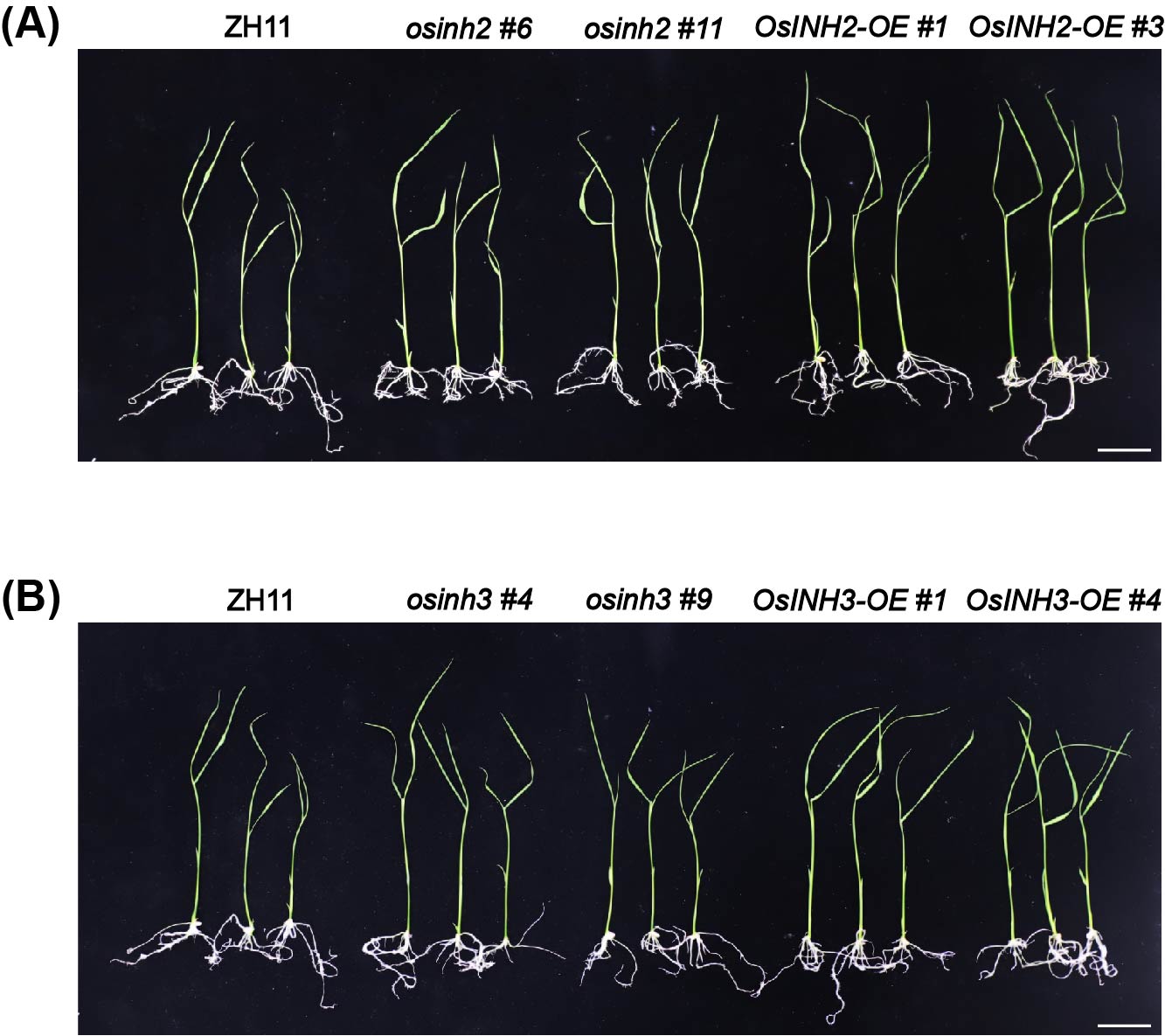

Supplement: SUPPLEMENTARY FIGURE 5 — Growth of OsINH2 and OsINH3 lines at the seedling stage. (A,B) Growth of OsINH2 and OsINH3 knockout and overexpression lines. Seedlings were grown on ½ MS media for 14th days and photographed. Scale bars, 2 cm. [file Image_5.JPEG]

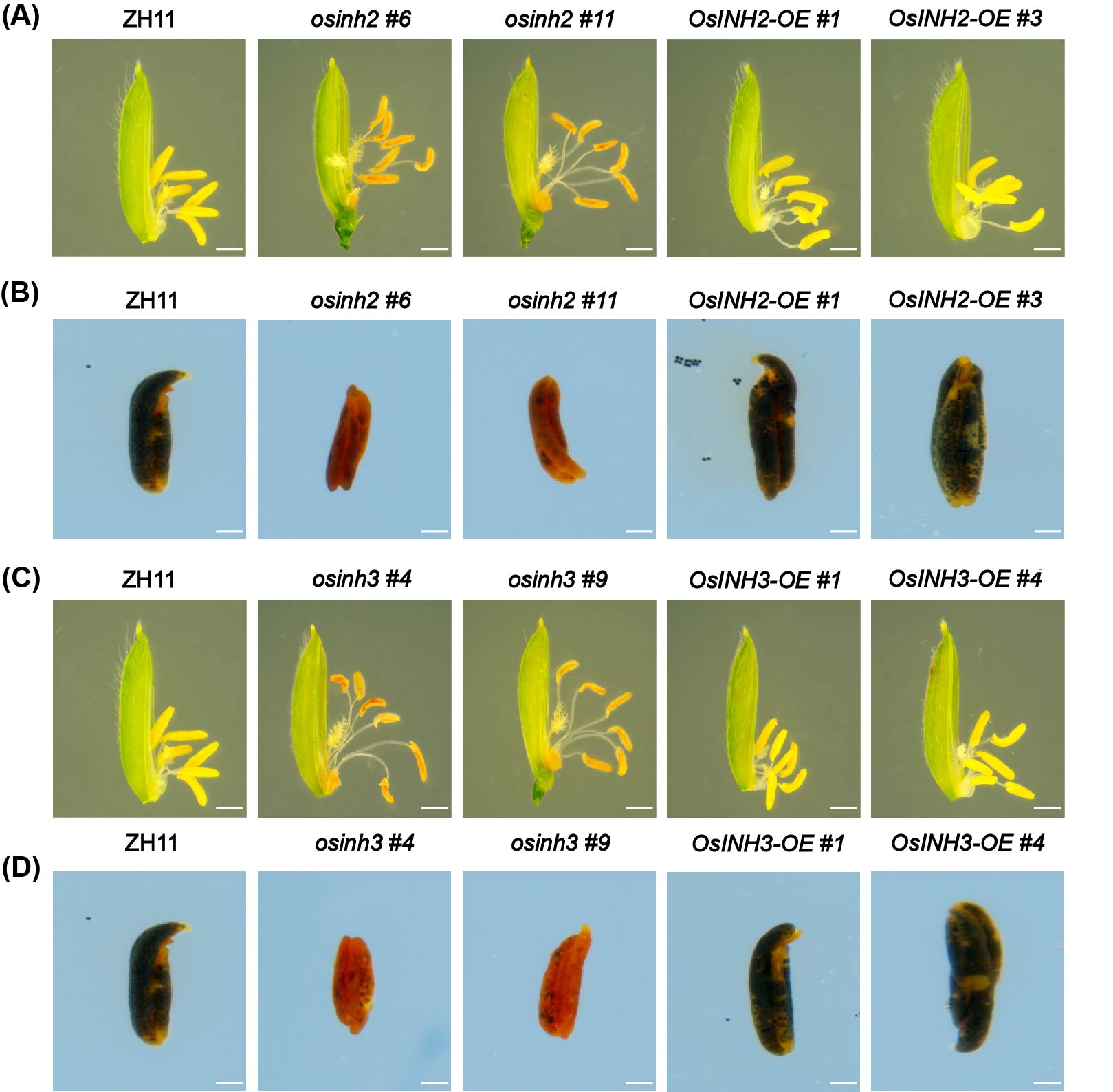

Supplement: SUPPLEMENTARY FIGURE 6 — Microscopic observation of flowers and anthers of OsINH2 and OsINH3 genotypes. (A) Flower phenotype of OsINH2 lines. (B) Stained anthers of OsINH2 genotypes. (C) Flower phenotype of OsINH3 lines. (D) Stained anthers of OsINH3 genotypes. Flowers enclosed in bracts were selected for microscopic observation. Anthers were stained by 1–2 drops of potassium iodide solution (KI). In (B,D) anthers represent dark blue (active) enclosed pollens, scale bars, 20 mm. [file Image_6.JPEG]

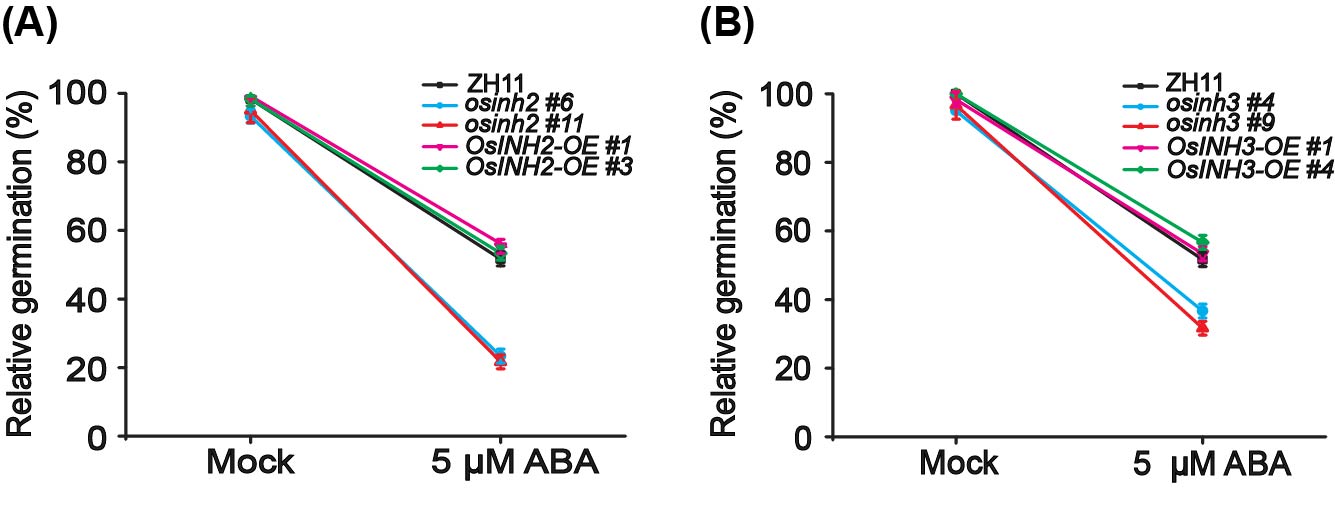

Supplement: SUPPLEMENTARY FIGURE 7 — Relative germination percentage of OsINH2 and OsINH3. (A,B) Relative germination percentage of OsINH2 and OsINH3 lines. Seeds were germinated on ½ MS media with and without ABA. The germination (%) was recorded on the 4th day. The experiment was repeated three times with similar results. [file Image_7.JPEG]

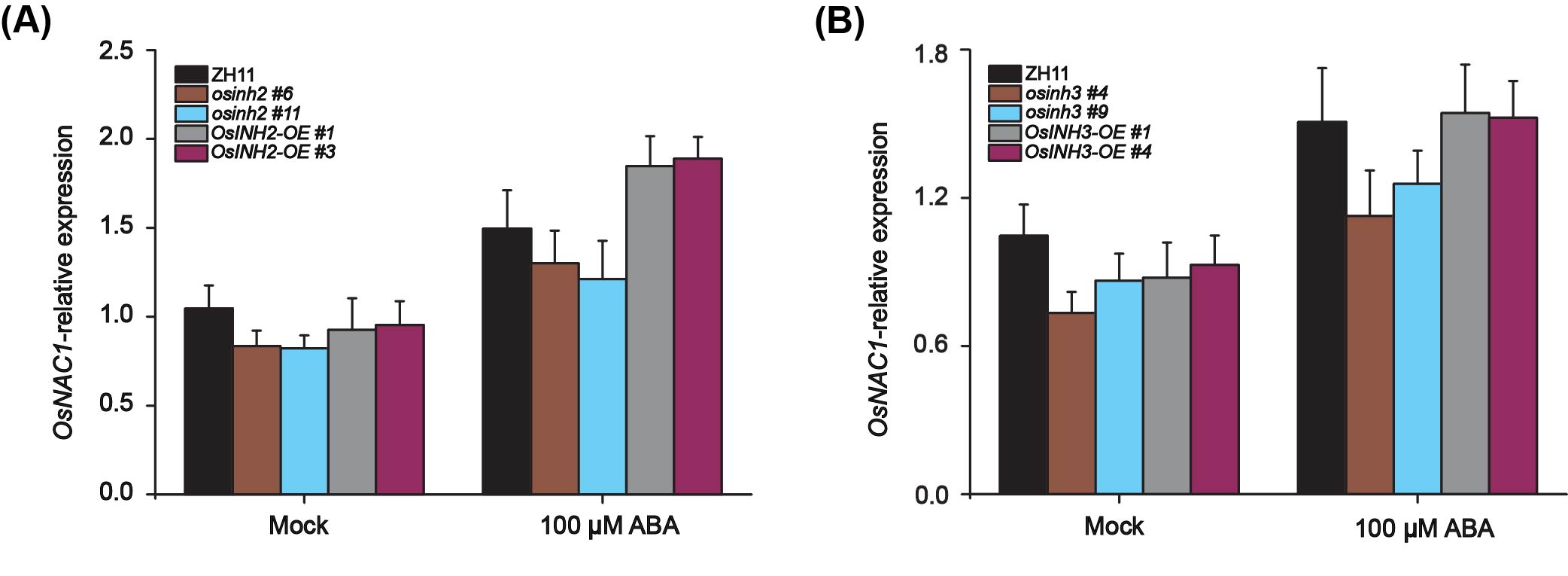

Supplement: SUPPLEMENTARY FIGURE 8 — Relative expression of OsNAC1 in OsINH2 and OsINH3 lines under ABA treatment. (A,B) Expression profile of OsNAC1 in OsINH2 and OsINH3 transgenic materials. Seeds were germinated on ½ MS media and one-week-old seedlings were transferred to nutrient solution and sprayed with 100 μM ABA at the three-leaf stage. Total RNA was extracted from leaves. Graph bars represent mean data. Error bars show ± SE in three repeats. The UBQ10 was used as a reference gene. The expression level was analyzed by student’s t-test at p < 0.05. [file Image_8.JPEG]
