# Supplementary material for: Rice protein phosphatase 1 regulatory subunits OsINH2 and OsINH3 participate actively in growth and adaptive responses under abscisic acid
Source: Front Plant Sci. 2022 Sep 7;13:990575. doi: 10.3389/fpls.2022.990575 (PMC9521630; doi:10.3389/fpls.2022.990575)
Supplement: Supplementary file 9 [file Data_Sheet_1.docx]

| **S. No** | **Gene Name** | **Gene ID** | **Forward/Reverse** | **Primer sequence (5’-3’)** |
| --- | --- | --- | --- | --- |
| **1** | *OsINH2* | LOC_Os05g23610 | Forward Primer | GGTGTGGAAATGCTGGACTG |
|  |  |  | Reverse Primer | GTCATTGTCACCAACGCGTC |
| **2** | *OsINH3* | LOC_Os05g18774 | Forward Primer | ACCAAGATTCGCCGCCC |
|  |  |  | Reverse Primer | TGACCTGGAAGGAGGGGAC |

**Supplementary Table 1. Primers used for identification of CRISPR/Cas9- mediated knockout lines.**

**Supplementary Table 2. Primers used for qRT-PCR**

| **S. No** | **Gene Name** | **Gene ID** | **Forward/Reverse** | **Primer sequence (5’-3’)** |
| --- | --- | --- | --- | --- |
| **1** | OsINH2 | LOC_Os05g23610 | Forward Primer | ATGATGCAGATGCCATGGAAC |
|  |  |  | Reverse Primer | CGCATCAGCTCCTTCACCT |
| **2** | OsINH3 | LOC_Os05g18774 | Forward Primer | GGCCGAAGAAGAAGGTGAC |
|  |  |  | Reverse Primer | AAGGGGACCTCCTTGTGGA |
| **3** | OsLEA3 | LOC4339480 | Forward Primer | AAGCAGAAGACCGCCGAGAC |
|  |  |  | Reverse Primer | GCTCTTCACCTGCTCACTCG |
| **4** | OsLIP19 | LOC4337699 | Forward Primer | TCGGCAGCGTCCTGGAGA |
|  |  |  | Reverse Primer | CAGAACTGGAAGGCGTCGG |
| **5** | OsP5CS1 | LOC4338979 | Forward Primer | GCTGACATGGATATGGCAAAAC |
|  |  |  | Reverse Primer | GTAAGGTCTCCATTGCATTGCA |
| **6** | OsNAC1 | LOC4334553 | Forward Primer | AGGCGCTCGTGTTCTACGC |
|  |  |  | Reverse Primer | CCCTGCTGCATCTTCTCCC |
| **7** | UBQ-10 | LOC_Os02g06640 | Forward Primer | TCTGATCTTCGCTGGCAA |
|  |  |  | Reverse Primer | CAAACTGGCTGATTACTGACC |

**Supplementary Table 3. List of genes accession number**

| **S No.** | **Gene Name** | **Gene Locus** |
| --- | --- | --- |
| **1** | OsINH2 | LOC_Os05g23610 |
| **2** | OsINH3 | LOC_Os05g18774 |
| **3** | OsTOPP1 | [LOC_Os08g35440](https://bioinformatics.psb.ugent.be/plaza/versions/plaza_v4_monocots/genes/view/LOC_Os08g35440) |
| **4** | OsTOPP2 | LOC-Os06g06880 |
| **5** | OsTOPP3 | LOC_Os02g57450 |
| **6** | OsTOPP4 | [LOC_Os03g16110](https://bioinformatics.psb.ugent.be/plaza/versions/plaza_v4_monocots/genes/view/LOC_Os03g16110) |
| **7** | OsTOPP5 | [LOC_Os01g24750](https://bioinformatics.psb.ugent.be/plaza/versions/plaza_v4_monocots/genes/view/LOC_Os01g24750) |
| **8** | OsSAPK1 | LOC_Os03g27280 |
| **9** | OsSAPK2 | LOC-Os07g42940 |
| **10** | OsSAPK3 | LOC_Os10g41490 |
| **11** | OsSAPK4 | [LOC_Os01g64970](https://bioinformatics.psb.ugent.be/plaza/versions/plaza_v4_monocots/genes/view/LOC_Os01g64970) |
| **12** | OsSAPK5 | LOC_Os04g59450 |
| **13** | OsSAPK6 | LOC_Os02g34600 |
| **14** | OsSAPK8 | [LOC_Os03g55600](https://bioinformatics.psb.ugent.be/plaza/versions/plaza_v4_monocots/genes/view/LOC_Os03g55600) |
| **15** | OsSAPK9 | [LOC_Os12g39630](https://bioinformatics.psb.ugent.be/plaza/versions/plaza_v4_monocots/genes/view/LOC_Os12g39630) |
